# Genome-Wide Linkage Disequilibrium in Nine-Spined Stickleback Populations

**DOI:** 10.1534/g3.114.013334

**Published:** 2014-08-12

**Authors:** Ji Yang, Takahito Shikano, Meng-Hua Li, Juha Merilä

**Affiliations:** * CAS Key Laboratory of Animal Ecology and Conservation Biology, Institute of Zoology, Chinese Academy of Sciences (CAS), Beijing 100101, China; † Ecological Genetics Research Unit, Department of Biosciences, FIN-00014 University of Helsinki, Finland

**Keywords:** genetic variation, linkage disequilibrium, microsatellite, *Pungitius pungitius*

## Abstract

Variation in the extent and magnitude of genome-wide linkage disequilibrium (LD) among populations residing in different habitats has seldom been studied in wild vertebrates. We used a total of 109 microsatellite markers to quantify the level and patterns of genome-wide LD in 13 Fennoscandian nine-spined stickleback (*Pungitius pungitius*) populations from four (*viz*. marine, lake, pond, and river) different habitat types. In general, high magnitude (*D*’ > 0.5) of LD was found both in freshwater and marine populations, and the magnitude of LD was significantly greater in inland freshwater than in marine populations. Interestingly, three coastal freshwater populations located in close geographic proximity to the marine populations exhibited similar LD patterns and genetic diversity as their marine neighbors. The greater levels of LD in inland freshwater compared with marine and costal freshwater populations can be explained in terms of their contrasting demographic histories: founder events, long-term isolation, small effective sizes, and population bottlenecks are factors likely to have contributed to the high levels of LD in the inland freshwater populations. In general, these findings shed new light on the patterns and extent of variation in genome-wide LD, as well as the ecological and evolutionary factors driving them.

During the processes of population differentiation and local adaptation, evolutionary forces of selection, drift, gene flow, and mutation jointly influence the structure and patterning of genetic variation in the genome. Ultimately, this influences the extent and strength of associations among different parts of the genome. Such genetic associations are reflected in nonrandom coinheritance of alleles at different loci, a phenomenon known as linkage disequilibrium (LD; Lewontin and Kojima 1960). Interest toward LD recently has been fueled by its fundamental role in determining the required marker density and feasibility of gene mapping approaches (Jorde 2000; Zondervan and Cardon 2004). Knowledge about the extent and magnitude of LD also has the potential to provide valuable insights into an organism’s evolutionary past (Nordborg and Tavaré 2002; Slatkin 2008). For instance, the degree and extent of genome-wide LD can help to identify population substructuring and demographic events such as bottlenecks and admixture (*e.g.*, Nei and Li 1973; Golding and Strobeck 1980). Similarly, patterns of local LD can help to uncover the history of mutation, gene conversion, and selection (*e.g.*, Karlin and Feldman 1970; Frisse *et al.* 2001). In this perspective, studies of LD also can be viewed as bridging evolutionary biology to genomics.

During the past few years, molecular markers across the whole genome have become available in many species, facilitating progress in quantifying the magnitude and patterns of genome-wide LD, for example in human (*e.g.*, Reich *et al.* 2001; Shifman *et al.* 2003), livestock (*e.g.*, Corbin *et al.* 2010; Badke *et al.* 2012; García-Gámez *et al.* 2012; Espigolan *et al.* 2013), crop (*e.g.*, Hao *et al.* 2011; Van Inghelandt *et al.* 2011; Delourme *et al.* 2013; Fang *et al.* 2013), and model species (*e.g.*, Mukai *et al.* 1971; Nordborg *et al.* 2002; Branca *et al.* 2011). However, the information about genome-wide LD in wild vertebrate populations remains limited to a few studies of mammals (*e.g.*, Hernandez *et al.* 2007; Laurie *et al.* 2007), birds (*e.g.*, Backström *et al.* 2006; Li and Merilä 2010; Kawakami *et al.* 2014), and fishes (*e.g.*, Hohenlohe *et al.* 2012). Yet, studies of LD in the wild are important, because they can address biological questions that are not approachable by use of laboratory or domestic populations. These include, for instance, mapping quantitative trait loci (QTL) or candidate genes for ecologically and environmentally important traits in the wild (*e.g.*, Slate 2005, 2013; Laurie *et al.* 2007; Ellegren and Sheldon 2008; Gratten *et al.* 2008; Slate *et al.* 2009), and disclosing the relative contributions of different factors like natural selection and demography shaping organism’s genome (*e.g.*, Cutter 2006). Furthermore, knowledge about interpopulation and interhabitat variation in genomic LD can be helpful in advancing our understanding of evolutionary processes in nature (Gould and Johnston 1972; Roesti *et al.* 2013). Several earlier studies have described differences in the degree and extent of LD among populations of humans (*e.g.*, Service *et al.* 2006), domestic animals (*e.g.*, Sutter *et al.* 2004; Badke *et al.* 2012), and cultivated plants (Hao *et al.* 2011; Fang *et al.* 2013). However, interpopulation comparisons of LD in wild vertebrates are scarce (but see: Li and Merilä 2011; Miller *et al.* 2011; Hohenlohe *et al.* 2012). Hence, more empirical studies are needed to advance our understanding of variation in the extent and magnitude of LD in the wild.

The nine-spined stickleback (*Pungitius pungitius*) is a small cold-water adapted fish with a circumpolar distribution in the northern hemisphere (Wootton 1976). Fennoscandian nine-spined stickleback populations have been derived from a common ancestral population and became established after the last glacial maximum (Shikano *et al.* 2010a; Teacher *et al.* 2011). They occur in both freshwater and marine habitats along the coastal areas of the White Sea and the Baltic Sea (Shikano *et al.* 2010a; Defaveri *et al.* 2012). Due to differing selection pressures among habitats, the species has undergone marked adaptive differentiation and, thus, shows pronounced morphological, physiological, and behavioral differentiation across habitat types (Merilä 2013). For instance, freshwater populations display reduced body armor (*e.g.*, Herczeg *et al.* 2010; Shikano *et al.* 2013), gigantism (*e.g.*, Herczeg *et al.* 2009), increased aggression (*e.g.*, Herczeg and Välimäki 2011), and divergent brain architecture (*e.g.*, Gonda *et al.* 2012) compared with marine populations. Earlier population genetic and phylogeographic studies (Shikano *et al.* 2010a; Teacher *et al.* 2011; Bruneaux *et al.* 2013) also suggest that postglacial recolonization and associated founder events have strongly affected the genetic variability and structure of current populations. Despite this progress in understanding local adaptation and differentiation among nine-spined stickleback populations (see also: Karhunen *et al.* 2014), possible differences in the extent and levels of genome-wide LD among populations and habitat types remain unknown.

The main aim of this study was to quantify and compare the patterns and extent of genome-wide LD in nine-spined stickleback populations from different habitats (*viz*. marine, river, lake, and pond). To this end, we used genotypic data on 109 microsatellite loci from 13 different nine-spined stickleback populations. Because isolated freshwater populations have very low levels of genetic variability (Shikano *et al.* 2010a; Bruneaux *et al.* 2013) and thus, are likely to have smaller effective population sizes and be more susceptible to stochastic demographic events than open and more genetically variable marine populations, we expected to find greater levels of genomic LD in freshwater compared with marine populations.

## Materials and Methods

### Study populations and samples

**Figure 1:**
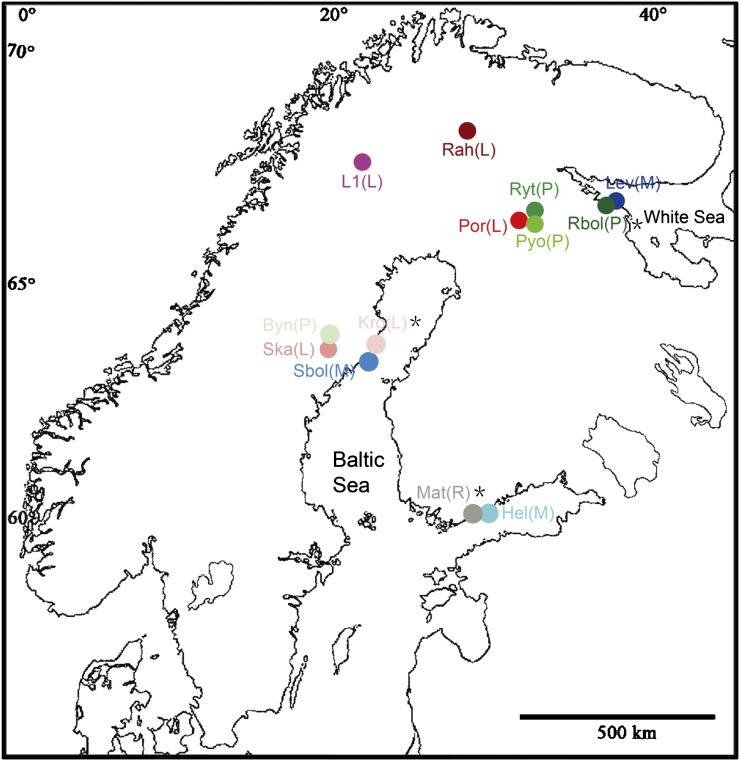
Map showing the locations of 13 nine-spined stickleback populations used in this study. The abbreviations of the populations are defined in Table 1. The letter in brackets stands for habitat type (M = marine; R = river; L = lake; *P* = pond). Asterisks indicate coastal freshwater populations.

A total of 312 nine-spined stickleback individuals (24 per population) from three marine and 10 freshwater populations were included in the analyses. The sampling sites covered a large part of the Fennoscandian area and encompassed a diverse array of habitats (*viz*. marine, river, lake, and pond populations; Figure 1 and Table 1). Marine fish were collected from the White Sea (Lev) and the Baltic Sea (Sbol and Hel), whereas freshwater fish were collected from one river (Mat), five lakes (Rah, L1, Por, Ska, and Kro) and four ponds (Ryt, Rbol, Pyo, and Byn; Figure 1). Three of the freshwater populations (Mat, Kro, and Rbol) located in close proximity to coastlines (Figure 1) were referred to as coastal freshwater populations, while the other seven freshwater populations (Rah, L1, Por, Ska, Ryt, Pyo, and Byn; Figure 1) were considered as inland freshwater populations.

**Table 1:** Sample information and genetic variation at 109 microsatellite loci in 13 nine-spined stickleback populations

Population	Habitat	*n*	*npl*	*A*	*A*r	*P*r	*H*_O_	*H*_E_	*F*_IS_ (95% CI)
Helsinki (Hel)	Marine	24	106	760	6.97	0.72	0.554	0.569	0.027 (−0.027−0.034)
Bölesviken (Sbol)	Marine	24	103	757	6.94	0.69	0.556	0.573	0.030 (−0.025−0.038)
Levin Navolok (Lev)	Marine	24	103	765	7.02	0.63	0.531	0.545	0.026 (−0.025−0.029)
Kroktjärnen (Kro)	Lake	24	103	647	5.94	0.44	0.562	0.574	0.021 (−0.024−0.020)
Västre-Skavträsket (Ska)	Lake	24	52	266	2.44	0.34	0.201	0.199	−0.012 (−0.088−0.019)
Iso-Porontima (Por)	Lake	24	90	397	3.64	0.20	0.300	0.320	0.064 (0.012−0.066)
Lake 1 (L1)	Lake	24	79	266	2.44	0.17	0.309	0.309	−0.002 (−0.064−0.015)
Rahajärvi (Rah)	Lake	24	89	524	4.81	0.37	0.358	0.368	0.030 (−0.041−0.046)
Bynastjärnen (Byn)	Pond	24	67	240	2.20	0.04	0.240	0.239	−0.004 (−0.073−0.019)
Pyöreälampi (Pyo)	Pond	24	33	164	1.50	0.03	0.084	0.085	0.002 (−0.092−0.047)
Bolotnoje (Rbol)	Pond	24	104	656	6.02	0.31	0.523	0.533	0.020 (−0.029−0.020)
Rytilampi (Ryt)	Pond	24	68	253	2.32	0.27	0.232	0.230	−0.009 (−0.081−0.016)
Matinoja (Mat)	River	24	103	507	4.65	0.24	0.522	0.530	0.015 (−0.040−0.023)

*n*, number of sampled individuals; *npl*, number of polymorphic loci; *A*, number of alleles; *A*r, allelic richness; *P*r, private allelic richness; *H*_O_, observed heterozygosity; *H*_E_, expected heterozygosity; *F*_IS_, departure from panmixia; CI, confidence interval.

### Molecular analyses

Total genomic DNA for the samples was extracted from fin clips using the phenol–chloroform method (Taggart *et al.* 1992) following proteinase K digestion. The same panel of 112 microsatellites as used by Shikano *et al.* (2010b) was used in all analyses. The genotyping data of the microsatellite markers for eight populations (Lev, Sbol, Hel, Mat, L1, Kro, Rbol, and Pyo) were taken from Shikano *et al.* (2010b,c), whereas the data for other five populations (Rah, Por, Ska, Ryt, and Byn) were produced in the present study (Supporting Information, File S1). Polymerase chain reactions (PCRs) were carried out using the QIAGEN multiplex PCR Kit (QIAGEN) in a reaction volume of 10 μL containing 1× QIAGEN multiplex PCR Master Mix, 0.5× Q-Solution, 2 pmol of each primer, and 10–20 ng of genomic DNA. The PCR amplifications were performed using the following cycle: initial activation at 95° for 15 min, followed by 30 s at 94°, 90 s at 53 or 55°, and 60 s at 72° for 30 cycles, ending with a final extension at 60° for 5 min. PCR products were resolved on a MegaBACE 1000 automated sequencer (Amersham Biosciences), and their sizes were determined with ET-ROX 550 size standard (Amersham Biosciences). Alleles were scored using FRAGMENT PROFILER 1.2 (Amersham Biosciences) with visual inspection and manual corrections.

### Population genetic analyses

Within-population observed heterozygosities (*H*_O_), expected heterozygosities (*H*_E_), inbreeding coefficient (*F*_IS_), and allele frequencies were calculated with FSTAT v2.9.3.2 (Goudet 2002). The proportion of rare alleles (allele frequency <5%) in each population was estimated using Microsoft Excel. Measures of allelic richness and private allelic richness for each population were calculated using HP-RARE (Kalinowski 2005), accounting for rarefaction.

Three approaches were used to investigate population genetic structure. First, pairwise *F*_ST_ among populations was calculated using GENETIX v4.03 (Belkhir *et al.* 2004), and the significance of *F*_ST_ values was evaluated via 10,000 permutations. Second, principal component analysis was performed at the individual level using the program GenAlex 6.501 (Peakall and Smouse 2006, 2012). Third, to assess the relative contributions of potential factors to population differentiation, a hierarchical analysis of molecular variance was performed using the program Arlequin v3.5 (Excoffier and Lischer 2010), based on three different grouping patterns of populations: habitat type I (marine, lake, pond, and river), habitat type II (marine and freshwater) and geographic proximity (Hel and Mat; Sbol and Kro; Ska and Byn; Por, Pyo, and Ryt; Rbol and Lev; L1; and Rah; see Figure 1). Statistical significance was assessed with 10,000 permutations. As population substructure tends to inflate LD (Nei and Li 1973; Pritchard and Przeworski 2001), we performed Bayesian clustering analyses in STRUCTURE v2.3.4 (Pritchard *et al.* 2000) to examine whether the observed high levels of LD (see the section *Results*) were due to within-population substructuring. We conducted three independent runs for each *K*-value ranging from 1 to 20. The admixture model and correlated allele frequencies model (Falush *et al.* 2003; Excoffier *et al.* 2005) were used, with 500,000 iterations after a 100,000 burn-in for each run. Also hidden family structure could amplify LD, and thus, we used Queller and Goodnight’s method (Queller and Goodnight 1989) implemented in program IDENTIX v1.1.5 (Belkhir *et al.* 2002) to estimate pairwise relatedness coefficient between individuals within each population.

Signatures of genetic bottlenecks were tested for each population using two methods. First, we used the heterozygosity excess method (Luikart *et al.* 1998) as implemented in the program Bottleneck v1.2.02 (Piry *et al.* 1999) to test for recent reductions in population size. We ran the program under the two-phased mutation model (TPM) with 90% single-step mutations. Statistical significance of the results was evaluated by 1000 iterations with a one-tailed Wilcoxon signed-rank test. Second, we used the *M*-ratio method (Garza and Williamson 2001) to detect historical population contractions (Garza and Williamson 2001; Williamson-Natesan 2005). Population-specific values of *M* (the number of alleles / the allele size range) and *M*_c_ (the critical value of *M*) were estimated using the programs M_P_VAL and CRITICAL_M (Garza and Williamson 2001), respectively. For each run, the simulations consisted of 10,000 iterations with the average mutation rate (*μ*) of 1.5 × 10^−4^ per generation (Shimoda *et al.* 1999), a TPM with 10% multistate change and 3.5 base steps for the mean size of multistep mutations (Garza and Williamson 2001). We tested three conservative values of theta (*θ* = 4*N*_e_*μ*) that equate to a prebottleneck effective population size (*N*_e_) of 1000, 5000, and 10,000 for the three marine and three coastal freshwater populations, and a prebottleneck *N*_e_ of 100, 500, and 1000 for the seven inland freshwater populations. The observed value of *M* was compared with the corresponding *M*_c_, and a lower value of *M* relative to *M*_c_ indicated a historical population bottleneck (Garza and Williamson 2001).

### Linkage map and haplotype phasing

**Figure 2:**
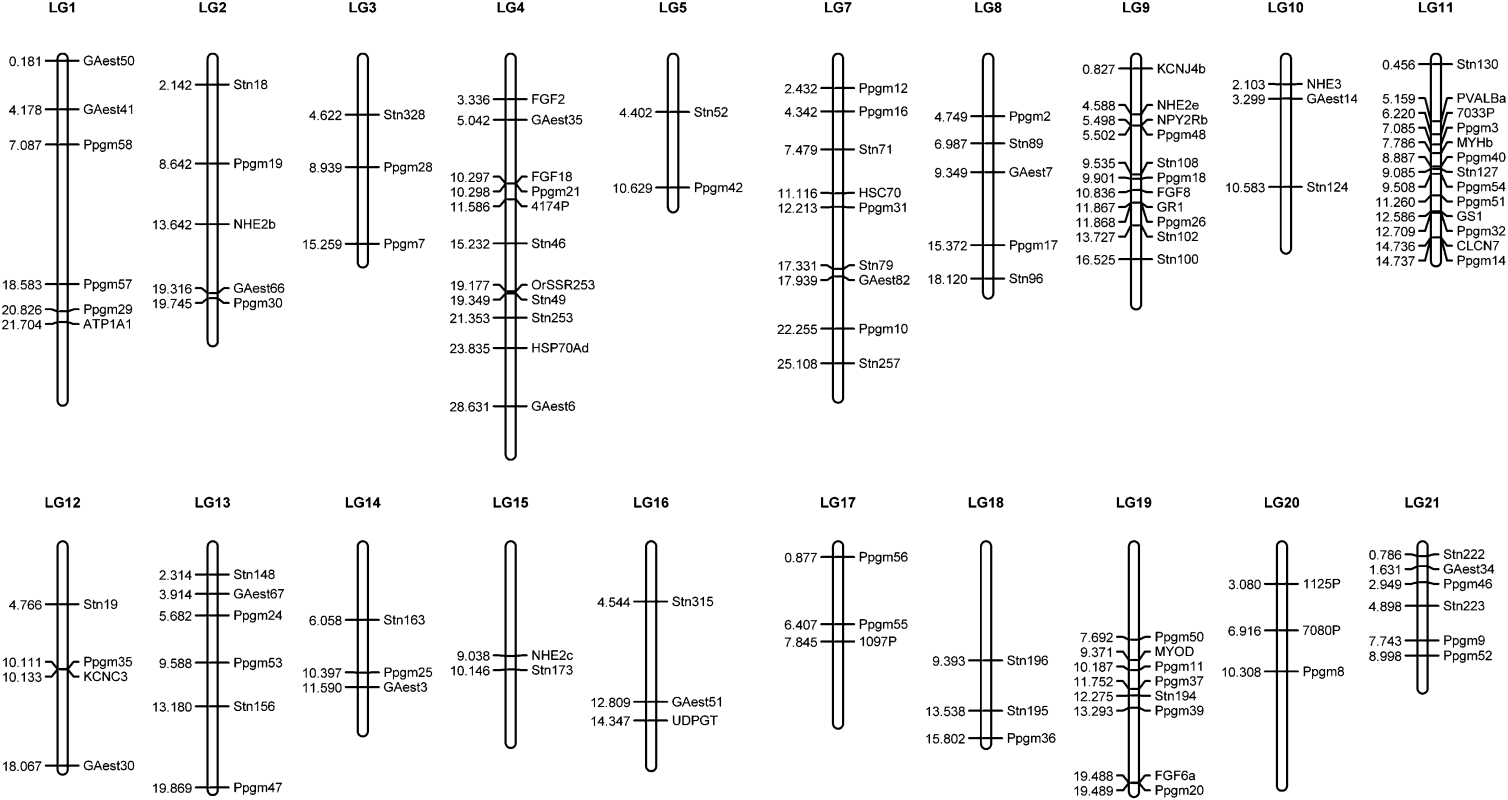
Genome-wide linkage map for nine-spined stickleback based on 109 microsatellite markers. Genomic distances (in Megabases, Mb) are listed on the left side of each linkage group (LG). All 109 loci were involved in linkage disequilibrium (LD) analyses.

Since nine-spined and three-spined sticklebacks (*Gasterosteus aculeatus*) have the same number (*n* = 21) of chromosomes (Chen and Reisman 1970) and syntenic locations of microsatellite loci are conserved between these two closely related species (Shapiro *et al.* 2009; Shikano *et al.* 2010c, 2013), we built the genomic distance-based (Mb) linkage map for the nine-spined stickleback through its homology with the three-spined genome assembly (http://www.ensembl.org/Gasterosteus_aculeatus/index.html). BLAST searches were performed to locate the 112 nine-spined stickleback microsatellite markers in the three-spined stickleback genome using the BLASTN tool in the Ensembl database. Initial searches were performed with the default conditions, and a locus was assigned to a genomic location if it provided a unique hit at E ≤ 1*e*^−10^. When a locus provided multiple matches at E ≤ 1*e*^−10^, it was unassigned unless the best hit had an E value at least 10 decimal places lower than the next best one. For ease of comparison, we numbered linkage groups (LGs) for the nine-spined stickleback linkage map in accordance with the syntenic LGs in the three-spined stickleback (Figure 2).

The gametic phase of haplotypes and missing genotypes were inferred from genotype data for each LG in each population and habitat type using a Bayesian statistical method as implemented in PHASE v2.1 (Stephens *et al.* 2001; Stephens and Scheet 2005). In each run, we chose the original model defined in Stephens *et al.* (2001), and set the number of iterations to 1000, thinning interval to 1 and a burn-in to 100. Ten independent runs were performed with different seeds to check for consistency between the results. We considered the PHASE results to be consistent when no less than eight runs gave the same inferred haplotypes, and in such case the consistent haplotypes were used in the subsequent calculations; otherwise, the haplotypes from the run with the highest average value for the goodness of fit statistics were used for the subsequent analyses (Stephens *et al.* 2001).

### LD analyses

Two different gametic LD measures, multiallelic *D*’ and *r^2^*, were used. The two LD estimates were derived from the standard measure of LD between two alleles at two different loci: *D_ij_* = *p*(A*_i_*B*_j_*) − *p*(A*_i_*)*p*(B*_j_*), where *p*(A*_i_*) is the frequency of allele A*_i_* at locus A, *p*(B*_j_*) is the frequency of allele B*_j_* at locus B, and *p*(A*_i_*B*_j_*) is the frequency of haplotype A*_i_*B*_j_* in the population.

Multiallelic *D*’ was estimated as (Lewontin 1964; Hedrick 1987):

$$D^{\prime }=\sum_{i=1}^{k}\sum_{j=1}^{l}p\left(A_{i}\right)p\left(B_{j}\right)\left|\frac{D_{ij}}{D_{ij}^{\max}}\right|$$

where *k* and *l* were the number of alleles for markers A and B, respectively, and

$$\begin{array}{c} D_{ij}^{\max}=\min\left[p\;\left(A_{i}\right)p\;\left(B_{j}\right),\;\left(1-p\;\left(A_{i}\right)\right)\left(1-p\;\left(B_{j}\right)\right)\right]\;\mathrm{when}\;D_{\mathrm{ij}}<0\;\mathrm{and}\\ D_{ij}^{\max}=\min\left[p\;\left(A_{i}\right)\left(1-p\;\left(B_{j}\right)\right),\;p\;\left(B_{j}\right)\left(1-p\;\left(A_{i}\right)\right)\right]\;\mathrm{when}\;D_{\mathrm{ij}}\geq 0. \end{array}$$

Multiallelic *r^2^* was estimated as (Hill and Robertson 1968):

$$r^{2}=\sum_{i=1}^{k}\sum_{j=1}^{l}\frac{D_{ij}^{2}}{p\left(A_{i}\right)\left(1-p\left(A_{i}\right)\right)p\left(B_{j}\right)\left(1-p\left(B_{j}\right)\right)}$$

We computed *D*’ and *r^2^* for all pairwise syntenic markers in each population and habitat type using the program PowerMarker v3.25 (Liu and Muse 2005). Pearson’s and Kendall’s correlation tests were performed to investigate the correlation between *D*’ and *r^2^* values within population or habitat. Because the measure *D*’ commonly has been used in studies of wild vertebrates (*e.g.*, Backström *et al.* 2006; Hohenlohe *et al.* 2012) and has more power to detect LD (Devlin and Risch 1995), it was used in the following analyses to facilitate comparison of our results with those of other studies. Logarithmic regression plots of *D*’ values of all syntenic pairwise markers against genomic distances (Mb) in each population and habitat type were generated in Microsoft Excel. The half-length of LD (Reich *et al.* 2001), *i.e.*, the distance at which it falls to 0.5, was evaluated.

Mann-Whitney *U*-tests (Mann and Whitney 1947) were used to assess the statistical significance of differences in *D*’ values between habitat types. Kruskal-Wallis tests (Kruskal and Wallis 1952) were used to assess the significance of differences in *D*’ values across all of the populations or among populations within the same habitat type. Partly different polymorphic markers were involved in different population-specific LD analyses (Table 1), hence the variation in marker distance between populations could potentially influence statistical significance tests of *D*’ values. In order to control for this, we used analysis of covariance (ANCOVA) in which population and habitat were treated as random and fixed factors, respectively, and associated *D*’ values were regarded as dependent variables, with physical distance between markers as a covariate. Furthermore, there were differences in marker density in different LGs. In order to corroborate the LD patterns observed in the genome-wide analyses, we examined LD patterns in four LGs with the greatest marker densities (*i.e.*, LGs 9, 11, 19, and 21; Figure 2) for each population. All statistical tests were conducted in SPSS 16.0 (SPSS Inc, Chicago, IL), and Bonferroni corrections (Rice 1989) were applied to adjust significance levels when multiple testing was involved.

To examine whether observed high levels of LD could be an artifact due to haplotype phasing, we also estimated the composite LD measure (Weir 1996) based on unphased genotypic data using the method described in Zaykin *et al.* (2008). In addition, to examine the effect of rare alleles (allele frequency <5%) on the levels of LD, we recalculated both haplotypic and composite LD measures with rare alleles excluded.

## Results

### Population genetic analyses

One-hundred nine microsatellite markers were successfully mapped to the three-spined stickleback genome. The basic indices of within-population genetic variability are given in Table 1. The number of polymorphic loci ranged from 33 (in Pyo) to 106 (in Hel) depending on the population. Allelic richness and expected heterozygosities (*H*_E_) estimated across all loci ranged from 1.50 (in Pyo) to 7.02 (in Lev), and from 0.085 (in Pyo) to 0.574 (in Kro), respectively (Table 1). Private allelic richness for each population ranged from 0.03 (in Pyo) to 0.72 (in Hel; Table 1). The marine (Hel, Sbol, and Lev) and coastal freshwater populations (Mat, Kro, and Rbol) had much greater genetic diversities (*H*_E_ = 0.530–0.574; Table 1) than the seven inland freshwater populations (Ska, Byn, Por, Pyo, Ryt, L1, and Rah; *H*_E_ = 0.085–0.368; Table 1). *F*_IS_ values and their 95% confidence intervals did not deviate significantly from zero in any of the populations (Table 1). A high proportion of rare alleles was observed within populations, ranging from 0.15 in Pyo to 0.53 in Hel (Table S1).

The extent of population differentiation as measured by *F*_ST_ among population pairs varied greatly (*F*_ST_ = 0.003−0.724), most of which were significant (52/78, *P* < 0.05/78 = 0.000641; Table S2). In general, *F*_ST_ values between inland freshwater populations were always greater than those between marine or coastal freshwater populations (Table S2). Principal component analysis revealed that the first and second axes accounted for 13.7% and 10.1% of variation in allele frequencies, respectively (Figure S1). The individuals from the inland freshwater populations clustered more tightly than those from the coastal freshwater and marine populations (Figure S1). Analysis of molecular variance analyses suggested that 7.4% of the total genetic variation was explained by geographic proximity (*P* < 0.001), whereas the factors of habitat type (marine *vs.* lake *vs.* pond *vs.* river, −1.9%, *P* > 0.05; marine *vs.* freshwater, −2.1%, *P* > 0.05; see Table 2) did not contribute to the patterns of genetic differentiation. Based on the value of *ΔK* (Evanno *et al.* 2005), STRUCTURE analyses indicated that the most probable *K* was nine (Figure S2). No substructure was found within any of the populations at both the optimal *K* value (*i.e.*, 9) and the maximum tested *K* value (*i.e.*, 20; Figure S2). Thus, population substructuring was unlikely to account for the observed high levels of LD. The estimated pairwise relatedness coefficients were generally small (*e.g.*, < 0.2) for 12 populations except Pyo (File S2), suggesting that most individuals should be unrelated; hence, family structure was not an explanation for the high LD values.

**Table 2:** Analysis of molecular variance in three different population groupings based on 109 microsatellite markers

Population Groups Defined	Components	Percentage of Variation
Four groups according to habitat type	Among groups	**−1.90**
	Among populations within groups	34.14***
	Within populations	67.76***
Seven groups according to geographic proximity	Among groups	7.35***
	Among populations within groups	25.65***
	Within populations	67.00***
Marine *vs.* freshwater populations	Among groups	**−2.05**
	Among populations within groups	33.75***
	Within populations	68.30***

*** *P* < 0.001. The percentage of genetic variation among groups is indicated by bold type.

A signal of recent population bottleneck was detected in only one population (L1; *P* = 0.03) under the TPM using the heterozygosity excess method. However, all populations except Pyo showed strong evidence for historical population bottlenecks using the *M*-ratio method, despite the differences in pre-bottleneck *N*_e_ (Table S3). Observed population-specific *M*-ratio values ranged from 0.670 to 0.898, and most (12/13, except Pyo) were lower than the corresponding *M*_c_ values (Table S3). It was unexpected that no bottleneck was detected in Pyo because this population had the lowest genetic diversity of all populations in this study (Table 1). However, this could be due to a small number of polymorphic markers (*n* = 33; Table 1) segregating in the population.

### Linkage map

Based on homologous positions in the three-spined stickleback genome, the 109 mapped microsatellites defined a total number of 20 LGs of the nine-spined stickleback (Figure 2). Two to 13markers were mapped to each of the LGs, but none of the markers mapped to LG6 of the three-spined stickleback (Figure 2). Based on the three-spined stickleback genome assembly, the average interval between adjacent markers was 2.738 Mb, with the smallest spacing of 0.001 Mb and the largest of 11.496 Mb. The median distance between adjacent markers was 2.004 Mb. With regard to different LGs, the average inter-marker distance ranged from 1.19 Mb in LG11 to 6.227 Mb in LG5. Inferred haplotypes from the program PHASE were largely consistent across the ten replicate runs, and approximately 90% of the total number of loci had phase probabilities of more than 0.8, indicating that the results were reliable.

### Genome-wide LD

Overall, the levels of syntenic LD as measured by *D*’ were relatively high (Table 3), but varied among the 13 populations (Kruskal-Wallis, χ^2^ = 100.20, *d.f*. = 12, *P* < 0.001; ANCOVA, *F*_12, 2911_ = 10.64, *P* < 0.001). When different habitat types were considered, lake (Mann-Whitney, *Z* = −4.99, *P* < 0.001; ANCOVA, *F*_1, 650_ = 15.37, *P* < 0.001), pond (Mann-Whitney, *Z* = −6.91, *P* < 0.001; ANCOVA, *F*_1, 646_= 45.75, *P* < 0.001), and river (Mann-Whitney, *Z* = −4.95, *P* < 0.001; ANCOVA, *F*_1, 646_ = 28.13, *P* < 0.001) habitats, showed significantly greater *D*’ values than the marine habitat. The greatest average *D*’ values were observed in the pond habitat (Table 3). There were no differences in *D*’ values among the different marine populations (*viz*. Hel, Sbol, Lev; Kruskal-Wallis, χ^2^ = 2.13, *d.f*. = 2, *P* = 0.34; ANCOVA, *F*_2, 937_ = 0.92, *P* = 0.40), but significant differences were found among the lake (*viz*. Kro, Ska, Por, L1, Rah; Kruskal-Wallis, χ^2^ = 64.94, *d.f*. = 4, *P* < 0.001; ANCOVA, *F*_4, 1049_ = 19.59, *P* < 0.001) and pond (*viz*. Byn, Pyo, Rbol, Ryt; Kruskal-Wallis, χ^2^ = 15.95, *d.f*. = 3, *P* < 0.001; ANCOVA, *F*_3, 609_ = 7.08, *P* < 0.001) populations. When restricting the comparisons to LGs with high density markers (LG9, LG11, LG19, LG21; 38 markers in total; Figure 2), the *D*’ values were similar to those obtained in the genome-wide analyses (all LGs; 109 markers in total; Figure 2) in 12 populations (Figure S3). This supports the view that the relatively low number of microsatellite markers used in this study can indeed yield information about general patterns of genome-wide LD. When LD was measured with *r^2^*, lower absolute values were observed (Table S4) compared with those of *D*’ (Table 3). However, *D*’ and *r^2^* values were positively and significantly correlated in most populations and habitat types (Table S5).

**Table 3:** Linkage disequilibrium estimate (*D*’) and associated estimation error for syntenic markers in 13 nine-spined stickleback populations and five habitat types (marine, lake, pond, river, and coastal freshwater) using 109 microsatellite markers

Data Set	Physical Distance Interval (Syntenic)					Overall (Syntenic)
	0−5 Mb	5.001−10 Mb	10.001−15 Mb	15.001−20 Mb	>20 Mb	
Hel (M)	0.557 (0.020)	0.549 (0.024)	0.500 (0.039)	0.492 (0.051)	0.649 (0.117)	0.544 (0.014)
Sbol (M)	0.557 (0.020)	0.553 (0.024)	0.534 (0.037)	0.578 (0.048)	0.519 (0.095)	0.553 (0.014)
Lev (M)	0.559 (0.020)	0.590 (0.026)	0.551 (0.037)	0.571 (0.043)	0.579 (0.103)	0.570 (0.014)
Kro (L)	0.509 (0.021)	0.504 (0.021)	0.445 (0.039)	0.442 (0.048)	0.373 (0.101)	0.491 (0.013)
Ska (L)	0.651 (0.050)	0.539 (0.050)	0.596 (0.090)	0.959 (0.042)	0.700 (0.174)	0.631 (0.033)
Por (L)	0.630 (0.031)	0.716 (0.035)	0.564 (0.068)	0.615 (0.088)	0.541 (0.147)	0.648 (0.021)
L1 (L)	0.551 (0.031)	0.428 (0.042)	0.536 (0.074)	0.453 (0.101)	0.471 (0.279)	0.506 (0.023)
Rah (L)	0.646 (0.027)	0.672 (0.029)	0.719 (0.051)	0.616 (0.082)	0.677 (0.053)	0.663 (0.018)
Byn (P)	0.553 (0.046)	0.636 (0.056)	0.628 (0.105)	0.608 (0.102)	0.767 (0.149)	0.605 (0.032)
Pyo (P)	0.804 (0.064)	0.710 (0.101)	0.579 (0.421)	1.000 (0.000)	—	0.781 (0.051)
Rbol (P)	0.537 (0.022)	0.599 (0.023)	0.526 (0.038)	0.455 (0.042)	0.522 (0.085)	0.551 (0.014)
Ryt (P)	0.631 (0.041)	0.604 (0.052)	0.577 (0.092)	0.521 (0.105)	0.768 (0.232)	0.612 (0.029)
Mat (R)	0.509 (0.021)	0.562 (0.027)	0.532 (0.041)	0.426 (0.043)	0.580 (0.122)	0.527 (0.015)
Marine (average*^a^*)	0.558 (0.001)	0.564 (0.013)	0.528 (0.015)	0.547 (0.028)	0.582 (0.038)	0.556 (0.008)
Lake (average*^a^*)	0.597 (0.037)	0.572 (0.069)	0.572 (0.058)	0.617 (0.121)	0.552 (0.080)	0.588 (0.047)
Pond (average*^a^*)	0.631 (0.070)	0.637 (0.029)	0.578 (0.024)	0.646 (0.141)	0.686 (0.082)	0.637 (0.058)
CF (average*^a^*)	0.518 (0.009)	0.555 (0.028)	0.501 (0.028)	0.441 (0.009)	0.492 (0.062)	0.523 (0.017)
Marine (combined*^b^*)	0.433 (0.017)	0.450 (0.021)	0.393 (0.033)	0.357 (0.035)	0.406 (0.082)	0.428 (0.012)
Lake (combined*^b^*)	0.507 (0.017)	0.486 (0.018)	0.469 (0.028)	0.451 (0.042)	0.503 (0.111)	0.491 (0.011)
Pond (combined*^b^*)	0.530 (0.022)	0.578 (0.023)	0.553 (0.037)	0.531 (0.046)	0.547 (0.069)	0.550 (0.014)
CF (combined*^b^*)	0.383 (0.017)	0.397 (0.021)	0.332 (0.020)	0.357 (0.039)	0.413 (0.094)	0.380 (0.011)
River	0.509 (0.021)	0.562 (0.027)	0.532 (0.041)	0.426 (0.043)	0.580 (0.122)	0.527 (0.015)

M, marine; L, lake; P, pond; R, river; CF, Coastal freshwater, including Kro, Rbol, and Mat. The population abbreviations are defined in Table 1. The value in the brackets is the estimation error associated to the mean *D*’ value, obtained by dividing the SD of *D*’ value by the square root of the number of marker pairs used to measure LD in each distance bin (Table S7).

a *D*′ value is directly obtained from the averaged *D*′ value of relevant populations.

b *D*′ value is calculated from the combined original haplotype data of relevant populations.

**Figure 3:**
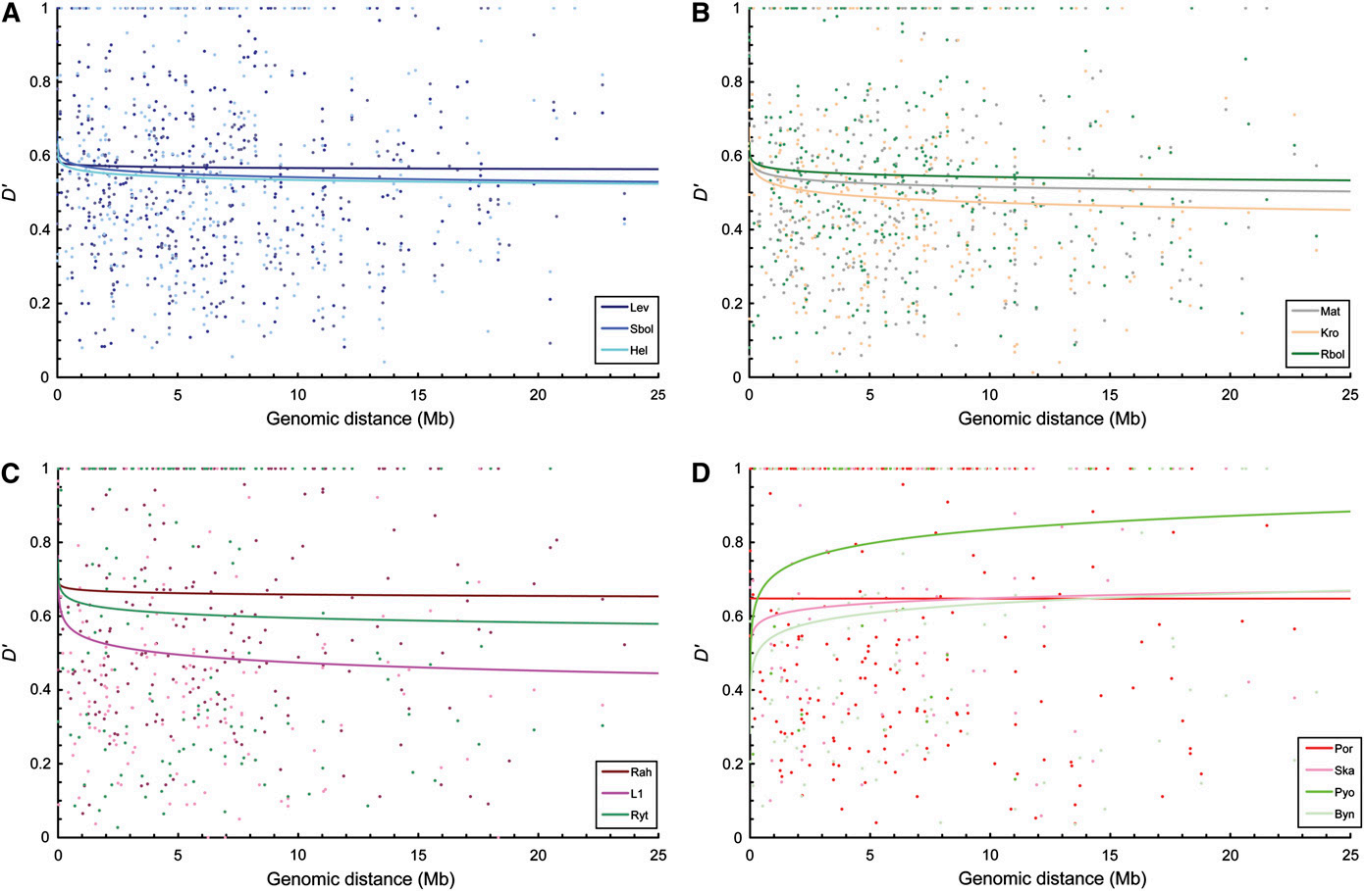
Observed linkage disequilibrium (LD, measured by *D*’) as a function of genomic distance (Megabases, Mb) between all syntenic markers in nine-spined stickleback populations using 109 microsatellite loci. (A) LD decay in three marine populations. (B) LD decay in three coastal freshwater populations. (C) LD decay in three inland freshwater populations with common decay pattern. (D) LD decay in four inland freshwater populations with unusual decay patterns. For population abbreviations, see Table 1.

**Figure 4:**
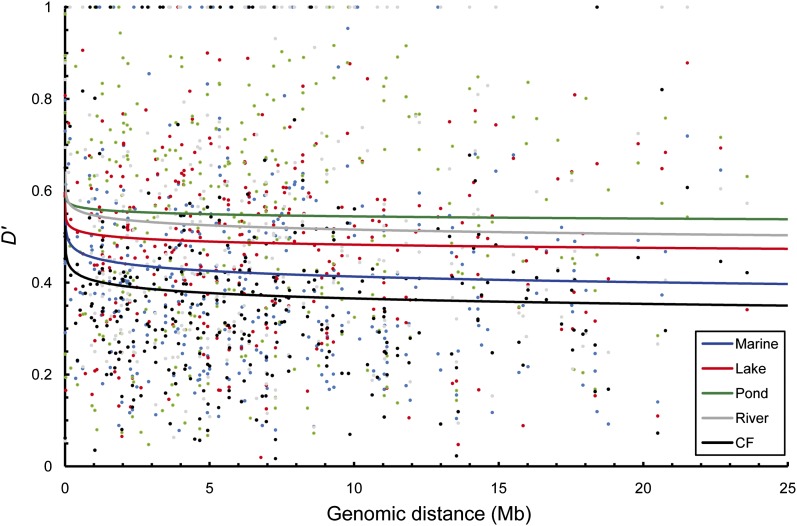
Linkage disequilibrium (LD, measured by *D*’) decay between all syntenic markers in five different habitat types (blue = marine populations, red = lake populations, green = pond populations, gray = river population, black = coastal freshwater [CF] populations). Combined population data of 109 microsatellite loci within the same habitat type were employed to estimate habitat-specific *D*’ values.

Comparison of the patterns of LD decay as a function of genomic distance revealed very weak and statistically nonsignificant (*R^2^* < 0.01, *P* > 0.05; Table S6 and Figure 3) correlations between *D*’ and genomic distance. With regard to LD decay in different habitats, the dataset of all marine populations combined or all freshwater populations combined showed higher correlations and shorter LD half-length compared with the combined lake or pond datasets (Figure 4 and Table S6). Interestingly, we found that the three coastal freshwater populations (Mat, Kro, Rbol; Figure 3B), which were geographically close to the marine populations (Hel, Sbol, Lev; Figure 3A), exhibited similar LD patterns as their marine neighbors, but deviated from the typical LD pattern in the inland freshwater populations (Figure 3C and Table 3). In addition, LD values increased slightly with genomic distance in three inland freshwater populations (Ska, Byn, Pyo; Figure 3D and Table S6), and the level of LD in Por was independent of genomic distance (Figure 3D and Table S6). This finding could be ascribable to stochasticity caused by the small number of marker pairs used to measure LD in each distance bin in these highly homozygous populations (Table S7).

The composite *D*’ and *r^2^* values were relatively high (Table S8) and comparable with the levels of haplotypic LD values (Table 3 and Table S4), indicating that observed high levels of LD were unlikely to be explainable as an effect of haplotype phasing. When the rare alleles were excluded, both haplotypic and composite *D*’ values were smaller, but the overall syntenic *D*’ value was still above 0.4 in almost all the populations (Table S8). On the contrary, both haplotypic and composite *r^2^* values became larger without the rare alleles (Table S8). Notably, irrespective of whether inferred haplotypic data or unphased genotypic data were used and whether the rare alleles were involved in the analyses or not, the findings about the LD patterns among habitat types (*i.e.*, Pond > Lake > Marine; Coastal freshwater is similar to Marine) based on combined data remained largely unchanged (Table 3, Table S4, and Table S8).

## Discussion

In general, low-to-moderate genetic diversity, strong genetic differentiation, and high levels of genome-wide LD were observed in Fennoscandian nine-spined stickleback populations. The extent and patterns of LD varied among populations and habitat types. Isolated and small freshwater populations tended to have greater LD compared with open marine populations. In the following, we will discuss these findings and their implications to our understanding of the factors influencing levels and extent of genomic LD in the wild.

Several recent studies have focused on fine-scale LD in commercially important fishes (*e.g.*, Hayes *et al.* 2006), whereas genome-wide levels of LD in wild fish populations remain largely unexplored, with few exceptions (*e.g.*, Hohenlohe *et al.* 2012; Roesti *et al.* 2013). We found high levels of LD in the studied nine-spined stickleback populations, and in this respect the results are comparable with those from the closely related three-spined stickleback (Mattern 2004; Mattern and Mclennan 2004), in which high magnitudes of LD were observed in both freshwater and marine populations (Hohenlohe *et al.* 2012). The high degree of LD in nine-spine sticklebacks did not come as a surprise in the view that earlier population genetic studies of this species (Shikano *et al.* 2010a; Teacher *et al.* 2011; Bruneaux *et al.* 2013) have suggested limited gene flow and low effective population sizes, both of which are factors expected to amplify genetic drift and thus the accumulation of LD (Service *et al.* 2006; Slatkin 2008; Charlesworth 2009). Likewise, demographic events such as founder effects and population bottlenecks can create high LD (*e.g.*, Nei and Li 1973; Zhang *et al.* 2004). In our case, the evidence for genetic bottlenecks in 12 of the 13 populations using *M*-ratio tests indicated that historical bottlenecks most probably have contributed to the high magnitude of genome-wide LD. Given that the stickleback populations studied here have been colonized after the last glacial maximum (<10,000 years ago), founder effects associated with postglacial recolonization also may account for the high LD. It should be noted that we have not taken recombination into account in our LD estimation due to its heterogeneity across the genome. Nevertheless, this should not affect the observed habitat or population differences in LD if the recombination hotspots are congruent in different populations, as has been reported for human populations (Conrad *et al.* 2006). One should also note that marker type can influence observed levels and extent of LD. For instance, microsatellite markers have more alleles per locus than SNP markers, and hence, they generally show higher levels of LD than SNPs (Chapman and Wijsman 1998). Consequently, the strong LD found here could partly be attributed to the high information content of microsatellites (Pritchard and Przeworski 2001). However, it is unlikely that this would be the sole explanation for the high levels of LD in nine-spined sticklebacks, especially in the view that this explanation cannot account for observed habitat or population differences in levels of LD. Other factors such as gene conversion, inversions and chromosome rearrangement could also have influenced the levels of LD in nine-spined sticklebacks, but the role of these factors remains to be investigated in future studies.

Despite the generally high magnitude of LD within populations, we also found significant differences in the levels and extent of LD between habitat types. The greatest levels of LD were observed in the seven inland freshwater populations, which was not unexpected as these are all population isolates that have been subject to substantial genetic drift due to initial founder effects, subsequent isolation and small effective population sizes. This drift has also led to reduced allelic diversity as reflected by low heterozygosities, low allelic richness, and overrepresentation of monomorphic microsatellite loci and rare alleles in these populations. This finding aligns well with those of earlier studies, which have shown that population isolates typically are characterized by low levels of genetic variation and high levels of LD (*e.g.*, Arcos-Burgos and Muenke 2002; Li and Merilä 2010). Interestingly, the patterns of LD and genetic variation in the three coastal freshwater populations were similar to those in the adjacent marine populations. Similar observations also were reported in an earlier study of Swedish nine-spined sticklebacks, which showed little genetic and morphological differentiation between marine and coastal lake populations in the Baltic Sea region (Herczeg *et al.* 2009; Mobley *et al.* 2011). One plausible explanation for these observations is that the coastal freshwater populations are influenced by admixture/gene flow from adjacent marine populations, or that they have only recently become isolated from the marine populations (Herczeg *et al.* 2009; Mobley *et al.* 2011).

Different metrics have been developed to measure the degree of LD, and we employed both *D*’ and *r^2^* estimators in this study. We found that the former yielded consistently higher values than the latter; such differences have also been reported in previous LD studies (*e.g.*, Shifman *et al.* 2003; García-Gámez *et al.* 2012; Espigolan *et al.* 2013). Several possible underlying factors could account for such differences, including large allele frequency differences between markers (*e.g.*, Ardlie *et al.* 2002; Wray *et al.* 2011) as was observed in this study (File S1). Likewise, the high proportion of rare alleles (allele frequency <5%; Table S1) and consequent loss of haplotypes in the populations may also yield high *D*’ values yet low *r^2^* values (Slatkin 2008; Purcell *et al.* 2009). Despite this discrepancy in absolute values of *D*’ and *r^2^*, the two estimators were positively correlated in our data (Table S5), and gave consistent LD patterns in inter-habitat comparisons (Table 3 and Table S4). Thus, conclusions drawn from *D*’ values are qualitatively similar to those obtained using *r^2^* values in respect to patterns of LD across habitat types.

Rare alleles (allele frequency <5%) tend to elevate *D*’ values (Teare *et al.* 2002); hence, they have often been eliminated from LD analyses. In our study, rare alleles were frequent in many populations, and this partly explains the high *D*’ values in this study. We believe that the inclusion of rare alleles in our LD analyses was reasonable on the following grounds: First, the overall syntenic *D*’ values remained relatively high (>0.4) in all of the 13 populations when the rare alleles were excluded. The differences in LD among habitat types (*i.e.*, Pond > Lake > Marine) remained unchanged even if the rare alleles were excluded. Second, rare variants can convey important information in genome-wide genetic studies (Dickson *et al.* 2010). Thus, given that the high proportion of rare alleles is an inherent characteristic of the nine-spined stickleback populations investigated here, ignoring them might bias the results. Third, given the demographic history of these populations, a high frequency of rare alleles is to be expected. Population genetics theory suggests that rare variants are likely to be recently derived alleles (Watterson and Guess 1977), and a large number of rare variants could derive from recent population expansions (Pritchard 2001; Gorlov *et al.* 2008). As for Fennoscandian nine-spined sticklebacks, earlier studies (Shikano *et al.* 2010a; Teacher *et al.* 2011; Bruneaux *et al.* 2013) indicated that populations inhabiting this region derived from ancestors in refugia from which the recolonization occurred approximately 10,000 years ago. Population expansions are very likely to have been involved in this re-establishment process, and thus, result in the large number of rare alleles in marine and coastal freshwater populations observed here. Previous studies have also indicated that inland freshwater populations have been established from marine populations recurrently (Teacher *et al.* 2011; Bruneaux *et al.* 2013). This finding, coupled with the fact that much genetic variation including rare alleles has been lost due to drift in inland isolates may explain why fewer rare alleles were observed in inland as compared to marine populations. In fact, within the same geographic region, an excess of rare alleles have also been observed in human (Reich *et al.* 2001) and Norway spruce (*Picea abies*) populations (Larsson *et al.* 2013).

Our findings of genomic LD and genetic variability have several important implications for gene mapping studies in nine-spined sticklebacks. First, given the high level of LD, a relatively small number of markers are required to cover a relatively large genomic region in QTL-mapping studies. Second, given the previous consideration, the mapping resolution will be relatively low because large genomic regions are likely to be inherited as linked clusters. Third, given the high frequency of rare alleles, nine-spined stickleback populations might prove to be suitable for rare variant mapping of complex traits. Nevertheless, although this study provides some preliminary insight on variation in LD across the nine-spined stickleback genome, one should bear in mind that the relatively low number of markers and their non-uniform distribution over the LGs and populations limit the inferences. Further exploration based on a larger number of markers, together with a high-density linkage map would pave the road for more refined inferences.

To sum up, the results provide the first investigation of genome-wide LD patterns in the nine-spined stickleback, and also one of the most extensive studies exploring patterns of habitat related variation in LD in wild vertebrates. In general, high levels of LD were observed in most of the analyzed populations, and more interestingly, higher levels of LD were detected in inland freshwater than in costal populations. This habitat patterning in the levels of LD matches what we discovered—and what has been known from earlier studies—about habitat-specific differences in demographic history and effective population size in these populations. The levels of LD uncovered in present study also suggest that studies seeking to disclose the genetic basis of phenotypic traits using QTL-mapping approaches may face challenges, especially in inland freshwater populations which are low in genetic variability and exhibit high levels of LD: the few polymorphic markers segregating in those populations are likely to be associated for long stretches of linked genes.

## Supplementary Material

Supporting Information
