# Supplementary material for: Genome-Wide Linkage Disequilibrium in Nine-Spined Stickleback Populations
Source: G3 (Bethesda). 2014 Aug 12;4(10):1919–29. doi: 10.1534/g3.114.013334 (PMC4199698; doi:10.1534/g3.114.013334)
Supplement: Supporting Information [file supp_4_10_1919__index.html]

Genome-Wide Linkage Disequilibrium in Nine-Spined Stickleback Populations — Supporting Information 

# Genome-Wide Linkage Disequilibrium in Nine-Spined Stickleback Populations

## Supporting Information for Yang *et al.*, 2014

**Files in this Data Supplement:**

- Supporting Information - Figures S1-S3, Files S1 and S3, and Tables S1-S8 (PDF, 1.2 MB)
- Table S6 - Logarithmic function describing the relationship between genome-wide LD (measured by *D'*) and genomic distance (Mb) together with the explanatory power (*R2*) and statistical significance (*P*) in 13 nine-spined stickleback populations and five habitat types (marine, lake, pond, river and coastal freshwater) using 109 microsatellite loci. (PDF, 60 KB)
- Table S7 - The number of microsatellite marker pairs in each distance bin (according to Table 3) for 13 nine-spined stickleback populations and five habitat types (marine, lake, pond, river and coastal freshwater). (PDF, 60 KB)
- Table S8 - Summary of linkage disequilibrium estimates (? S.E.) for syntenic markers in nine-spined stickleback populations and habitat types with both haplotypic and genotypic data and either including or excluding rare alleles using 109 microsatellite markers (PDF, 68 KB)
- Figure S1 - Principal component analysis (PCA) of 312 nine-spined stickleback individuals from 13 different populations based on 109 microsatellite loci (PDF, 140 KB)
- Figure S2 - STRUCTURE outputs for joint analysis of the 13 nine-spined stickleback populations (PDF, 924 KB)
- Figure S3 - (PDF,Histograms showing comparisons of mean *D'* values based on all 109 microsatellite markers (genome-wide) and on 38 microsatellite markers in four LGs with highest density of markers in 13 nine-spined stickleback populations 132 KB)
- Table S1 - The number and proportion of rare alleles (allele frequency < 0.05) in 13 nine-spined stickleback populations (PDF, 57 KB)
- Table S2 - Matrix of pairwise *FST* estimates (lower diagonal) and their statistical significance (upper diagonal) between 13 nine-spined stickleback populations based on 109 microsatellite loci (PDF, 62 KB)
- Table S3 - Results of bottleneck tests in 13 nine-spined stickleback populations form software Bottleneck under TPM mutation model with a one-tailed Wilcoxon signed-rank tests (P values) and from *M*-ratio tests (*M* and *Mc* values) using 109 microsatellite markers (PDF, 65 KB)
- Table S4 - Linkage disequilibrium estimates (*r2* ? S.E.) for syntenic markers in nine-spined stickleback populations and habitat types using 109 microsatellite markers (PDF, 61 KB)
- Table S5 - Results of Pearson's and Kendall's correlation tests between *D'* and *r2* values in nine-spined stickleback populations and habitat types (PDF, 58 KB)
- File S1 - Genotype data (.txt, 241 KB)
- File S2 - Pairwise relatedness coefficients (.txt, 43 KB)
