# Supplementary material for: Genome-Wide Linkage Disequilibrium in Nine-Spined Stickleback Populations
Source: G3 (Bethesda). 2014 Aug 12;4(10):1919–29. doi: 10.1534/g3.114.013334 (PMC4199698; doi:10.1534/g3.114.013334)
Supplement: Supporting Information [file supp_g3.114.013334_013334SI.pdf]

G3 Genes | Genomes | Genetics

Genome-wide linkage disequilibrium in nine-spined stickleback populations

Ji Yang,[*] Takahito Shikano,[†] Meng-Hua Li,[*,1] and Juha Merilä[†]

[*]CAS Key Laboratory of Animal Ecology and Conservation Biology, Institute of Zoology, Chinese Academy of Sciences (CAS), Beijing 100101, China

[†]Ecological Genetics Research Unit, Department of Biosciences, P.O. Box 65, FIN-00014 University of Helsinki, Finland

[1]Corresponding author: Meng-Hua Li, CAS Key Laboratory of Animal Ecology and Conservation Biology, Institute of Zoology, Chinese Academy of Sciences (CAS), Beichen West Road No. 1-5, Beijing 100101, China. Tel: +86-10-64806336; Fax: +86-10-64806336; E-mail: menghua.li@ioz.ac.cn

DOI: 10.1534/g3.114.013334

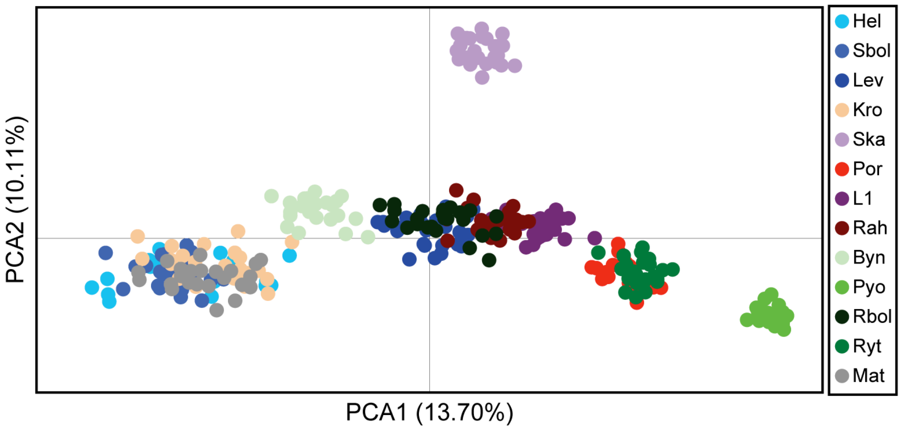

Figure S1 Principal component analysis (PCA) of 312 nine-spined stickleback individuals from 13 different populations based on 109 microsatellite loci. Small colored circles represent individuals and the used colors correspond to those in Fig 1. The population abbreviations are defined in Table 1.

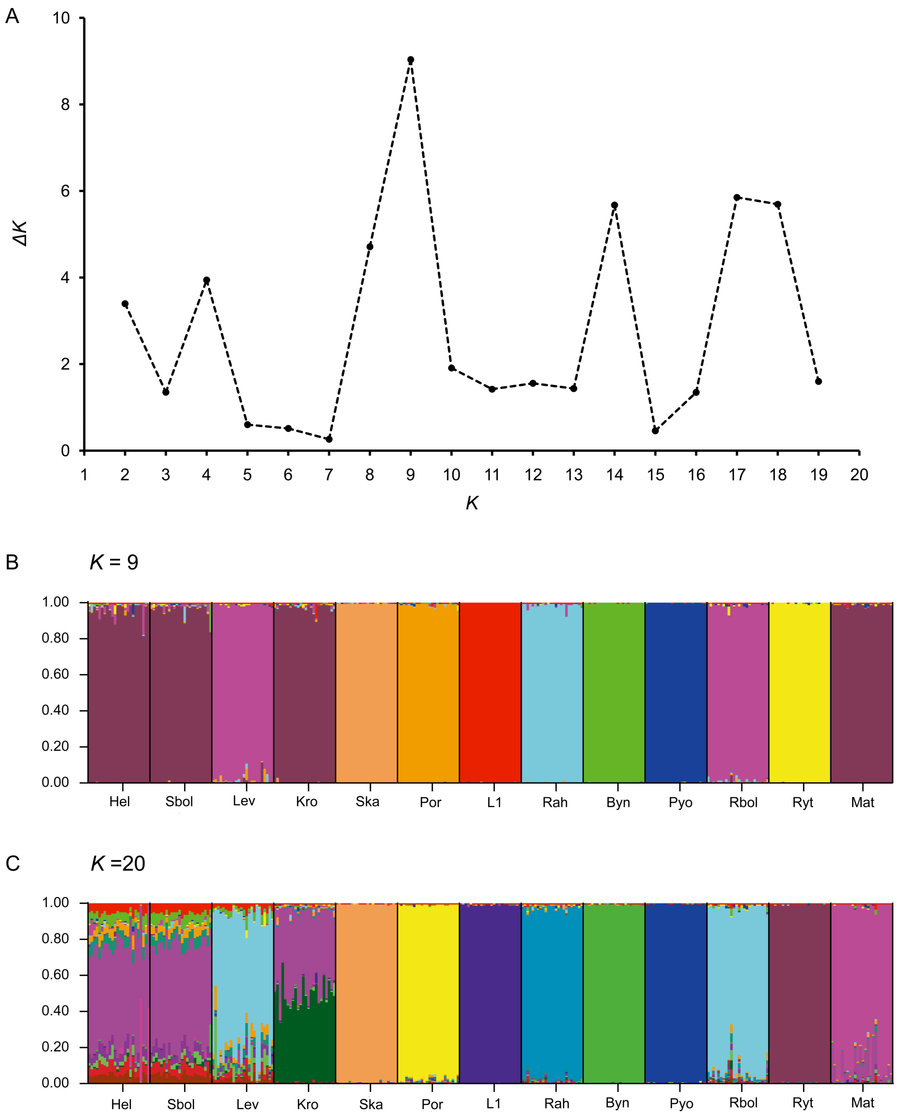

Figure S2 STRUCTURE outputs for joint analysis of the 13 nine-spined stickleback populations. (A) The maximum value of *ΔK* indicates the most likely number of genetic clusters (*K*) was nine. (B) Individual membership bar plot of the optimal nine genetic clusters. (C) Individual membership bar plot of the maximum tested 20 genetic clusters. The genotype of each individual is represented by a thin vertical bar representing the membership proportions in each of the genetic clusters. Each colour stands for a genetic cluster. No substructure was found within any of the populations at both *K* = 9 and *K* = 20. The population abbreviations are defined in Table 1.

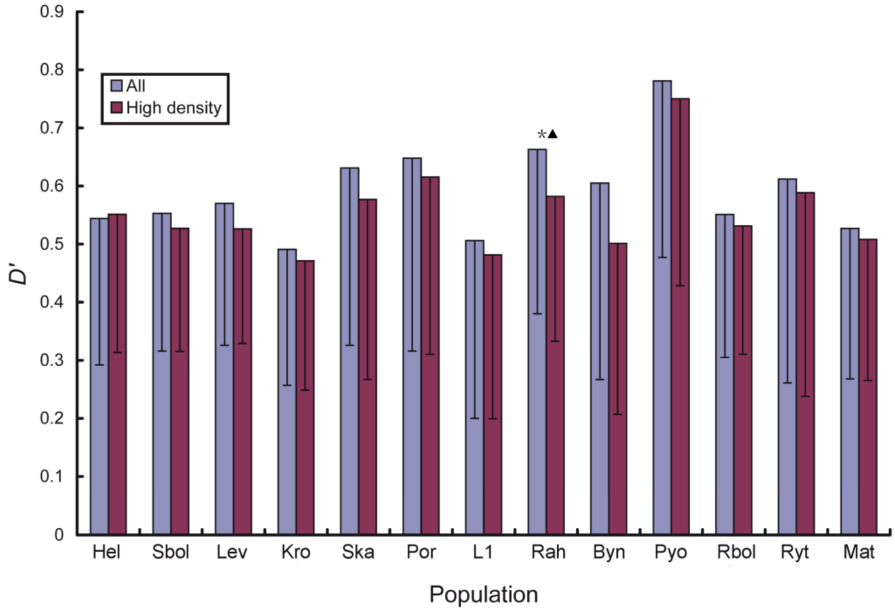

Figure S3 Histograms showing comparisons of mean *D'* values based on all 109 microsatellite markers (genome-wide) and on 38 microsatellite markers in four LGs with highest density of markers in 13 nine-spined stickleback populations. The line within vertical bar represents standard deviation of the mean value. Significant differences in *D'* values are indicated by asterisk (Mann-Whitney U tests, $P < 0.05$) and triangle (ANCOVAs, $P < 0.05$).

Table S1 The number and proportion of rare alleles (allele frequency < 0.05) in 13 nine-spined stickleback populations using 109 microsatellite markers.

Population	Total number of alleles	The number of rare alleles	Proportion of rare alleles
Hel (M)	760	399	0.53
Sbol (M)	757	392	0.52
Lev (M)	765	395	0.52
Kro (L)	647	267	0.41
Ska (L)	266	45	0.17
Por (L)	397	159	0.40
L1 (L)	266	68	0.26
Rah (L)	524	245	0.47
Byn (P)	240	39	0.16
Pyo (P)	164	25	0.15
Rbol (P)	656	299	0.46
Ryt (P)	253	62	0.25
Mat (R)	507	146	0.29

M, marine; L, lake; P, pond; R, river. The population abbreviations are defined in Table 1.

Table S2 Matrix of pairwise F_{ST} estimates (lower diagonal) and their statistical significance (upper diagonal) between 13 nine-spined stickleback populations based on 109 microsatellite loci.

Populations	Hel	Sbol	Lev	Kro	Ska	Por	L1	Rah	Byn	Pyo	Rbol	Ryt	Mat
Hel	—	NS	*	*	NS	NS	*	NS	*	*	*	*	*
Sbol	0.003	—	NS	NS	NS	NS	*	NS	*	*	*	*	*
Lev	0.100	0.103	—	*	NS	*	*	NS	*	*	*	*	*
Kro	0.020	0.017	0.096	—	NS	*	*	NS	*	*	*	*	*
Ska	0.376	0.372	0.316	0.359	—	NS	NS	NS	NS	NS	NS	NS	NS
Por	0.278	0.279	0.227	0.265	0.521	—	*	NS	*	*	*	*	*
L1	0.291	0.296	0.241	0.288	0.527	0.405	—	NS	*	*	*	*	*
Rah	0.243	0.248	0.195	0.239	0.475	0.390	0.388	—	NS	*	NS	*	NS
Byn	0.326	0.324	0.339	0.336	0.589	0.486	0.523	0.477	—	*	*	*	*
Pyo	0.455	0.455	0.410	0.449	0.724	0.548	0.617	0.573	0.703	—	*	*	*
Rbol	0.110	0.113	0.018	0.105	0.312	0.236	0.254	0.190	0.346	0.413	—	*	*
Ryt	0.352	0.362	0.314	0.357	0.604	0.454	0.492	0.428	0.571	0.679	0.314	—	*
Mat	0.044	0.043	0.120	0.057	0.398	0.304	0.311	0.278	0.347	0.472	0.130	0.371	—

NS, not significant; *Statistical significance at Bonferroni adjusted ($P < 0.000641$) alpha level of $P = 0.05$. The population abbreviations are defined in Table 1.

Table S3 Results of bottleneck tests in 13 nine-spined stickleback populations form software Bottleneck under TPM mutation model with a one-tailed Wilcoxon signed-rank tests (*P* values) and from *M*-ratio tests (*M* and M_c values) using 109 microsatellite markers.

Population	TPM	*M*	M_c
Hel	1.00	0.693	0.874*/0.792*/0.755*
Sbol	1.00	0.736	0.874*/0.792*/0.755*
Lev	1.00	0.753	0.874*/0.792*/0.755*
Kro	0.92	0.714	0.874*/0.792*/0.755*
Ska	0.89	0.847	0.906*/0.884*/0.861*
Por	1.00	0.797	0.916*/0.893*/0.871*
L1	0.03*	0.705	0.914*/0.891*/0.869*
Rah	1.00	0.780	0.915*/0.893*/0.872*
Byn	0.42	0.806	0.911*/0.889*/0.866*
Pyo	0.96	0.898	0.896/0.872/0.849
Rbol	1.00	0.735	0.874*/0.791*/0.755*
Ryt	0.69	0.772	0.911*/0.889*/0.867*
Mat	0.47	0.670	0.874*/0.792*/0.755*

*$P < 0.05$. The three M_c values from left to right correspond to the tests of a pre-bottleneck N_e of 1,000, 5,000, 10,000 for the three marine (Hel, Sbol, Lev) and three coastal freshwater (Kro, Rbol, Mat) populations, and 100, 500 and 1000 for the seven inland freshwater populations (Ska, Por, L1, Rah, Byn, Pyo, Rbol, Ryt), respectively.

Table S4 Linkage disequilibrium estimates (r^2 ± S.E.) for syntenic markers in nine-spined stickleback populations and habitat types using 109 microsatellite markers.

Data Set	Physical distance interval (Syntenic)					Overall
	0-5 Mb	5-10 Mb	10-15 Mb	15-20 Mb	>20 Mb	(Syntenic)
Hel (M)	0.031 (0.038)	0.032 (0.034)	0.028 (0.024)	0.024 (0.017)	0.037 (0.013)	0.030 (0.033)
Sbol (M)	0.035 (0.042)	0.033 (0.038)	0.033 (0.033)	0.029 (0.014)	0.020 (0.010)	0.033 (0.037)
Lev (M)	0.026 (0.018)	0.038 (0.066)	0.032 (0.032)	0.041 (0.030)	0.028 (0.018)	0.032 (0.043)
Kro (L)	0.033 (0.028)	0.035 (0.042)	0.026 (0.022)	0.022 (0.013)	0.022 (0.011)	0.032 (0.032)
Ska (L)	0.037 (0.032)	0.048 (0.036)	0.040 (0.041)	0.059 (0.046)	0.022 (0.012)	0.041 (0.035)
Por (L)	0.030 (0.032)	0.030 (0.057)	0.021 (0.027)	0.035 (0.034)	0.034 (0.042)	0.030 (0.042)
L1 (L)	0.052 (0.062)	0.062 (0.158)	0.085 (0.148)	0.034 (0.028)	0.011 (0.011)	0.056 (0.107)
Rah (L)	0.036 (0.057)	0.034 (0.094)	0.037 (0.043)	0.033 (0.038)	0.082 (0.065)	0.036 (0.069)
Byn (P)	0.035 (0.035)	0.066 (0.097)	0.077 (0.121)	0.034 (0.031)	0.015 (0.009)	0.050 (0.076)
Pyo (P)	0.045 (0.044)	0.131 (0.307)	0.008 (0.008)	0.013 (0.004)	—	0.065 (0.167)
Rbol (P)	0.029 (0.026)	0.045 (0.084)	0.034 (0.033)	0.027 (0.014)	0.029 (0.012)	0.035 (0.054)
Ryt (P)	0.046 (0.062)	0.049 (0.144)	0.036 (0.046)	0.047 (0.035)	0.013 (0.010)	0.046 (0.094)
Mat (R)	0.041 (0.049)	0.038 (0.042)	0.046 (0.051)	0.031 (0.022)	0.035 (0.022)	0.040 (0.045)
Marine (average[a])	0.031 (0.005)	0.034 (0.003)	0.031 (0.003)	0.031 (0.009)	0.028 (0.009)	0.032 (0.002)
Lake (average[a])	0.038 (0.009)	0.042 (0.013)	0.042 (0.025)	0.037 (0.014)	0.034 (0.028)	0.039 (0.010)
Pond (average[a])	0.039 (0.008)	0.073 (0.040)	0.039 (0.029)	0.030 (0.014)	0.019 (0.009)	0.049 (0.012)
CF (average[a])	0.034 (0.006)	0.039 (0.005)	0.035 (0.010)	0.027 (0.005)	0.029 (0.007)	0.036 (0.004)
Marine (combined[b])	0.014 (0.017)	0.016 (0.024)	0.015 (0.017)	0.012 (0.013)	0.010 (0.008)	0.014 (0.019)
Lake (combined[b])	0.035 (0.043)	0.035 (0.066)	0.036 (0.044)	0.034 (0.031)	0.021 (0.024)	0.035 (0.051)
Pond (combined[b])	0.057 (0.073)	0.069 (0.087)	0.068 (0.088)	0.085 (0.129)	0.042 (0.058)	0.064 (0.084)
CF (combined[b])	0.024 (0.043)	0.020 (0.022)	0.026 (0.023)	0.018 (0.011)	0.014 (0.010)	0.022 (0.033)
River	0.041 (0.049)	0.038 (0.042)	0.046 (0.051)	0.031 (0.022)	0.035 (0.022)	0.040 (0.045)

M, marine; L, lake; P, pond; R, river; CF, Coastal freshwater, including Kro, Rbol and Mat. The population abbreviations are defined in Table 1.

[a]r^2 value is obtained from the averaged r^2 value of relevant populations.

[b]r^2 value is calculated from the combined original haplotype data of relevant populations.

Table S5 Results of Pearson's and Kendall's correlation tests between *D'* and r^2 values in nine-spined stickleback populations and habitat types.

Population	Pearson's correlation coefficient	Kendall's correlation coefficient
Hel (M)	0.178**	0.277**
Sbol (M)	0.180**	0.175**
Lev (M)	0.265**	0.263**
Kro (L)	0.291**	0.307**
Ska (L)	0.160	0.175*
Por (L)	0.117	0.105*
L1 (L)	0.317**	0.371**
Rah (L)	0.149*	0.081
Byn (P)	0.244**	0.265**
Pyo (P)	0.106	-0.146
Rbol (P)	0.231**	0.279**
Ryt (P)	0.176*	0.218**
Mat (R)	0.263**	0.282**
Marine (combined data)	0.172**	0.195**
Lake (combined data)	0.274**	0.234**
Pond (combined data)	0.449**	0.417**
CF (combined data)	0.286**	0.304**
River	0.263**	0.282**

**P* < 0.05, ***P* < 0.01. M, marine; L, lake; P, pond; R, river; CF, Coastal freshwater, including Kro, Rbol and Mat. The population abbreviations are defined in Table 1.

Table S6 Logarithmic function describing the relationship between genome-wide LD (measured by *D'*) and genomic distance (Mb) together with the explanatory power (R^2) and statistical significance (*P*) in 13 nine-spined stickleback populations and five habitat types (marine, lake, pond, river and coastal freshwater) using 109 microsatellite loci.

Population	Logarithmic function	R^2	*P*
Hel (M)	*y* = -0.0113Ln(*x*) + 0.5603	0.0038	0.2357
Sbol (M)	*y* = -0.0134Ln(*x*) + 0.5728	0.0062	0.1668
Lev (M)	*y* = -0.0034Ln(*x*) + 0.5747	0.0004	0.74
Kro (L)	*y* = -0.022Ln(*x*) + 0.524	0.0168	0.0173*
**Ska (L)**	*y* = 0.0205Ln(*x*) + 0.6013	0.0072	0.4387
Por (L)	*y* = -0.0001Ln(*x*) + 0.6477	0.0000	0.9913
L1 (L)	*y* = -0.0315Ln(*x*) + 0.5463	0.0182	0.0817
Rah (L)	*y* = -0.0054Ln(*x*) + 0.671	0.0007	0.7054
**Byn (P)**	*y* = 0.0382Ln(*x*) + 0.5463	0.0217	0.116
**Pyo (P)**	*y* = 0.0538Ln(*x*) + 0.7105	0.0387	0.2575
Rbol (P)	*y* = -0.0101Ln(*x*) + 0.5659	0.0034	0.3011
Ryt (P)	*y* = -0.0173Ln(*x*) + 0.6348	0.0063	0.3342
Mat (R)	*y* = -0.0135Ln(*x*) + 0.5467	0.0054	0.2037
Marine	*y* = -0.0178Ln(*x*) + 0.4543	0.0129	0.0338*
Lake	*y* = -0.0099Ln(*x*) + 0.5053	0.005	0.2067
Pond	*y* = -0.0067Ln(*x*) + 0.56	0.0015	0.5276
River	*y* = -0.0135Ln(*x*) + 0.5467	0.0054	0.2037
Coastal freshwater	*y* = -0.01Ln(*x*) + 0.404	0.013	0.0386*

Population name in bold type indicates the population has an increasing trend. *$P < 0.05$. M, marine; L, lake; P, pond; R, river. The population abbreviations are defined in Table 1.

Table S7 The number of microsatellite marker pairs in each distance bin (according to Table 3) for 13 nine-spined stickleback populations and five habitat types (marine, lake, pond, river and coastal freshwater).

Data Set	Physical distance interval (Syntenic)					Overall
	0-5 Mb	5-10 Mb	10-15 Mb	15-20 Mb	>20 Mb	(Syntenic)
Hel (M)	144	109	50	20	7	330
Sbol (M)	129	101	49	20	7	306
Lev (M)	131	102	45	20	7	305
Kro (L)	136	103	46	19	5	309
Ska (L)	42	21	14	4	4	85
Por (L)	106	81	30	16	6	239
L1 (L)	93	52	16	9	3	173
Rah (L)	112	81	34	17	5	249
Byn (P)	47	36	16	10	6	115
Pyo (P)	20	10	2	3	0	35
Rbol (P)	137	105	46	20	7	315
Ryt (P)	73	48	16	9	3	149
Mat (R)	136	107	45	20	7	315
Marine	146	111	50	20	7	334
Lake	139	107	46	20	7	319
Pond	137	105	46	20	7	315
River	136	107	45	20	7	315
Coastal freshwater	145	113	46	20	7	331

M, marine; L, lake; P, pond; R, river. The population abbreviations are defined in Table 1.

Table S8 Summary of linkage disequilibrium estimates (± S.E.) for syntenic markers in nine-spined stickleback populations and habitat types with both haplotypic and genotypic data and either including or excluding rare alleles using 109 microsatellite markers.

Data Set	Hap (*D'*, MAF 0.05)	Hap (r^2, MAF 0.05)	Comp (*D'*)	Comp (*D'*, MAF 0.05)	Comp (r^2)	Comp (r^2, MAF 0.05)
Hel (M)	0.410 (0.219)	0.044 (0.044)	0.693 (0.191)	0.468 (0.233)	0.206 (0.059)	0.281 (0.133)
Sbol (M)	0.410 (0.200)	0.051 (0.055)	0.678 (0.204)	0.482 (0.234)	0.206 (0.064)	0.290 (0.141)
Lev (M)	0.487 (0.255)	0.048 (0.072)	0.685 (0.183)	0.491 (0.242)	0.205 (0.057)	0.280 (0.135)
Kro (L)	0.411 (0.222)	0.042 (0.041)	0.595 (0.203)	0.414 (0.199)	0.204 (0.062)	0.248 (0.099)
Ska (L)	0.495 (0.273)	0.048 (0.035)	0.559 (0.250)	0.405 (0.232)	0.208 (0.085)	0.240 (0.119)
Por (L)	0.447 (0.295)	0.038 (0.036)	0.681 (0.269)	0.424 (0.269)	0.197 (0.098)	0.245 (0.153)
L1 (L)	0.446 (0.289)	0.067 (0.122)	0.461 (0.237)	0.369 (0.205)	0.223 (0.121)	0.244 (0.140)
Rah (L)	0.489 (0.257)	0.053 (0.091)	0.729 (0.223)	0.504 (0.250)	0.204 (0.105)	0.297 (0.158)
Byn (P)	0.512 (0.325)	0.062 (0.088)	0.474 (0.281)	0.335 (0.217)	0.186 (0.107)	0.199 (0.123)
Pyo (P)	0.706 (0.331)	0.084 (0.212)	0.625 (0.350)	0.450 (0.363)	0.199 (0.173)	0.227 (0.214)
Rbol (P)	0.450 (0.230)	0.048 (0.071)	0.641 (0.207)	0.475 (0.227)	0.209 (0.062)	0.271 (0.136)
Ryt (P)	0.421 (0.293)	0.046 (0.058)	0.525 (0.297)	0.356 (0.213)	0.206 (0.117)	0.222 (0.128)
Mat (R)	0.443 (0.233)	0.046 (0.053)	0.563 (0.221)	0.405 (0.187)	0.212 (0.081)	0.236 (0.093)
Marine (average[a])	0.436 (0.044)	0.048 (0.004)	0.685 (0.008)	0.480 (0.012)	0.206 (0.001)	0.284 (0.006)
Lake (average[a])	0.458 (0.035)	0.050 (0.011)	0.605 (0.105)	0.423 (0.050))	0.207 (0.010)	0.255 (0.024)
Pond (average[a])	0.522 (0.128)	0.060 (0.018)	0.566 (0.080)	0.404 (0.069)	0.200 (0.010)	0.230 (0.030)
CF (average[a])	0.435 (0.021)	0.045 (0.003)	0.600 (0.039)	0.431 (0.038)	0.208 (0.004)	0.252 (0.018)
Marine (combined[b])	0.300 (0.183)	0.024 (0.033)	0.683 (0.166)	0.347 (0.195)	0.123 (0.032)	0.206 (0.121)
Lake (combined[b])	0.451 (0.207)	0.060 (0.071)	0.707 (0.154)	0.491 (0.195)	0.148 (0.060)	0.269 (0.118)
Pond (combined[b])	0.574 (0.264)	0.124 (0.141)	0.688 (0.176)	0.608 (0.256)	0.182 (0.078)	0.349 (0.184)
CF (combined[b])	0.306 (0.148)	0.028 (0.038)	0.623 (0.188)	0.324 (0.178)	0.130 (0.038)	0.189 (0.091)
River	0.443 (0.233)	0.046 (0.053)	0.563 (0.221)	0.405 (0.187)	0.212 (0.081)	0.236 (0.093)

M, marine; L, lake; P, pond; R, river; CF, Coastal freshwater, including Kro, Rbol and Mat. Hap, haplotypic LD measures; Comp, Composite LD measures.

MAF 0.05 = minor alleles with frequency less than 5% were excluded from the LD analyses. The population abbreviations are defined in Table 1.

[a]*D'* or r^2 value is obtained from the averaged *D'* or r^2 value of relevant populations.

[b]*D'* or r^2 value is calculated from the combined original haplotype (for Hap) or genotype (for Comp) data of relevant populations.

File S1 and S2

Genotype data and Pairwise relatedness coefficients are available for download at http://www.g3journal.org/lookup/suppl/doi: 10.1534/g3.114.013334/-/DC1.
