# Supplementary material for: Genome-Wide Linkage Disequilibrium in Nine-Spined Stickleback Populations
Source: G3 (Bethesda). 2014 Aug 12;4(10):1919–29. doi: 10.1534/g3.114.013334 (PMC4199698; doi:10.1534/g3.114.013334)
Supplement: Supporting Information [file supp_g3.114.013334_TableS6.pdf]

Table S6 Logarithmic function describing the relationship between genome-wide LD (measured by *D'*) and genomic distance (Mb) together with the explanatory power (R^2) and statistical significance (*P*) in 13 nine-spined stickleback populations and five habitat types (marine, lake, pond, river and coastal freshwater) using 109 microsatellite loci.

Population	Logarithmic function	R^2	*P*
Hel (M)	*y* = -0.0113Ln(*x*) + 0.5603	0.0038	0.2357
Sbol (M)	*y* = -0.0134Ln(*x*) + 0.5728	0.0062	0.1668
Lev (M)	*y* = -0.0034Ln(*x*) + 0.5747	0.0004	0.74
Kro (L)	*y* = -0.022Ln(*x*) + 0.524	0.0168	0.0173*
**Ska (L)**	*y* = 0.0205Ln(*x*) + 0.6013	0.0072	0.4387
Por (L)	*y* = -0.0001Ln(*x*) + 0.6477	0.0000	0.9913
L1 (L)	*y* = -0.0315Ln(*x*) + 0.5463	0.0182	0.0817
Rah (L)	*y* = -0.0054Ln(*x*) + 0.671	0.0007	0.7054
**Byn (P)**	*y* = 0.0382Ln(*x*) + 0.5463	0.0217	0.116
**Pyo (P)**	*y* = 0.0538Ln(*x*) + 0.7105	0.0387	0.2575
Rbol (P)	*y* = -0.0101Ln(*x*) + 0.5659	0.0034	0.3011
Ryt (P)	*y* = -0.0173Ln(*x*) + 0.6348	0.0063	0.3342
Mat (R)	*y* = -0.0135Ln(*x*) + 0.5467	0.0054	0.2037
Marine	*y* = -0.0178Ln(*x*) + 0.4543	0.0129	0.0338*
Lake	*y* = -0.0099Ln(*x*) + 0.5053	0.005	0.2067
Pond	*y* = -0.0067Ln(*x*) + 0.56	0.0015	0.5276
River	*y* = -0.0135Ln(*x*) + 0.5467	0.0054	0.2037
Coastal freshwater	*y* = -0.01Ln(*x*) + 0.404	0.013	0.0386*

Population name in bold type indicates the population has an increasing trend. *$P < 0.05$. M, marine; L, lake; P, pond; R, river. The population abbreviations are defined in Table 1.
