# Supplementary material for: Genome-Wide Linkage Disequilibrium in Nine-Spined Stickleback Populations
Source: G3 (Bethesda). 2014 Aug 12;4(10):1919–29. doi: 10.1534/g3.114.013334 (PMC4199698; doi:10.1534/g3.114.013334)
Supplement: Supporting Information [file supp_g3.114.013334_TableS7.pdf]

Table S7 The number of microsatellite marker pairs in each distance bin (according to Table 3) for 13 nine-spined stickleback populations and five habitat types (marine, lake, pond, river and coastal freshwater).

Data Set	Physical distance interval (Syntenic)					Overall (Syntenic)
	0-5 Mb	5-10 Mb	10-15 Mb	15-20 Mb	>20 Mb	
Hel (M)	144	109	50	20	7	330
Sbol (M)	129	101	49	20	7	306
Lev (M)	131	102	45	20	7	305
Kro (L)	136	103	46	19	5	309
Ska (L)	42	21	14	4	4	85
Por (L)	106	81	30	16	6	239
L1 (L)	93	52	16	9	3	173
Rah (L)	112	81	34	17	5	249
Byn (P)	47	36	16	10	6	115
Pyo (P)	20	10	2	3	0	35
Rbol (P)	137	105	46	20	7	315
Ryt (P)	73	48	16	9	3	149
Mat (R)	136	107	45	20	7	315
Marine	146	111	50	20	7	334
Lake	139	107	46	20	7	319
Pond	137	105	46	20	7	315
River	136	107	45	20	7	315
Coastal freshwater	145	113	46	20	7	331

M, marine; L, lake; P, pond; R, river. The population abbreviations are defined in Table 1.
