# Supplementary material for: Genome-Wide Linkage Disequilibrium in Nine-Spined Stickleback Populations
Source: G3 (Bethesda). 2014 Aug 12;4(10):1919–29. doi: 10.1534/g3.114.013334 (PMC4199698; doi:10.1534/g3.114.013334)
Supplement: Supporting Information [file supp_g3.114.013334_TableS8.pdf]

Table S8 Summary of linkage disequilibrium estimates (± S.E.) for syntenic markers in nine-spined stickleback populations and habitat types with both haplotypic and genotypic data and either including or excluding rare alleles using 109 microsatellite markers.

Data Set	Hap (*D'*, MAF 0.05)	Hap (r^2, MAF 0.05)	Comp (*D'*)	Comp (*D'*, MAF 0.05)	Comp (r^2)	Comp (r^2, MAF 0.05)
Hel (M)	0.410 (0.219)	0.044 (0.044)	0.693 (0.191)	0.468 (0.233)	0.206 (0.059)	0.281 (0.133)
Sbol (M)	0.410 (0.200)	0.051 (0.055)	0.678 (0.204)	0.482 (0.234)	0.206 (0.064)	0.290 (0.141)
Lev (M)	0.487 (0.255)	0.048 (0.072)	0.685 (0.183)	0.491 (0.242)	0.205 (0.057)	0.280 (0.135)
Kro (L)	0.411 (0.222)	0.042 (0.041)	0.595 (0.203)	0.414 (0.199)	0.204 (0.062)	0.248 (0.099)
Ska (L)	0.495 (0.273)	0.048 (0.035)	0.559 (0.250)	0.405 (0.232)	0.208 (0.085)	0.240 (0.119)
Por (L)	0.447 (0.295)	0.038 (0.036)	0.681 (0.269)	0.424 (0.269)	0.197 (0.098)	0.245 (0.153)
L1 (L)	0.446 (0.289)	0.067 (0.122)	0.461 (0.237)	0.369 (0.205)	0.223 (0.121)	0.244 (0.140)
Rah (L)	0.489 (0.257)	0.053 (0.091)	0.729 (0.223)	0.504 (0.250)	0.204 (0.105)	0.297 (0.158)
Byn (P)	0.512 (0.325)	0.062 (0.088)	0.474 (0.281)	0.335 (0.217)	0.186 (0.107)	0.199 (0.123)
Pyo (P)	0.706 (0.331)	0.084 (0.212)	0.625 (0.350)	0.450 (0.363)	0.199 (0.173)	0.227 (0.214)
Rbol (P)	0.450 (0.230)	0.048 (0.071)	0.641 (0.207)	0.475 (0.227)	0.209 (0.062)	0.271 (0.136)
Ryt (P)	0.421 (0.293)	0.046 (0.058)	0.525 (0.297)	0.356 (0.213)	0.206 (0.117)	0.222 (0.128)
Mat (R)	0.443 (0.233)	0.046 (0.053)	0.563 (0.221)	0.405 (0.187)	0.212 (0.081)	0.236 (0.093)
Marine (average[a])	0.436 (0.044)	0.048 (0.004)	0.685 (0.008)	0.480 (0.012)	0.206 (0.001)	0.284 (0.006)
Lake (average[a])	0.458 (0.035)	0.050 (0.011)	0.605 (0.105)	0.423 (0.050))	0.207 (0.010)	0.255 (0.024)
Pond (average[a])	0.522 (0.128)	0.060 (0.018)	0.566 (0.080)	0.404 (0.069)	0.200 (0.010)	0.230 (0.030)
CF (average[a])	0.435 (0.021)	0.045 (0.003)	0.600 (0.039)	0.431 (0.038)	0.208 (0.004)	0.252 (0.018)
Marine (combined[b])	0.300 (0.183)	0.024 (0.033)	0.683 (0.166)	0.347 (0.195)	0.123 (0.032)	0.206 (0.121)
Lake (combined[b])	0.451 (0.207)	0.060 (0.071)	0.707 (0.154)	0.491 (0.195)	0.148 (0.060)	0.269 (0.118)
Pond (combined[b])	0.574 (0.264)	0.124 (0.141)	0.688 (0.176)	0.608 (0.256)	0.182 (0.078)	0.349 (0.184)
CF (combined[b])	0.306 (0.148)	0.028 (0.038)	0.623 (0.188)	0.324 (0.178)	0.130 (0.038)	0.189 (0.091)
River	0.443 (0.233)	0.046 (0.053)	0.563 (0.221)	0.405 (0.187)	0.212 (0.081)	0.236 (0.093)

M, marine; L, lake; P, pond; R, river; CF, Coastal freshwater, including Kro, Rbol and Mat. Hap, haplotypic LD measures; Comp, Composite LD measures.

MAF 0.05 = minor alleles with frequency less than 5% were excluded from the LD analyses. The population abbreviations are defined in Table 1.

[a]*D'* or r^2 value is obtained from the averaged *D'* or r^2 value of relevant populations.

[b]*D'* or r^2 value is calculated from the combined original haplotype (for Hap) or genotype (for Comp) data of relevant populations.
