# Supplementary material for: Genome-Wide Linkage Disequilibrium in Nine-Spined Stickleback Populations
Source: G3 (Bethesda). 2014 Aug 12;4(10):1919–29. doi: 10.1534/g3.114.013334 (PMC4199698; doi:10.1534/g3.114.013334)
Supplement: Supporting Information [file supp_g3.114.013334_FigureS1.pdf]

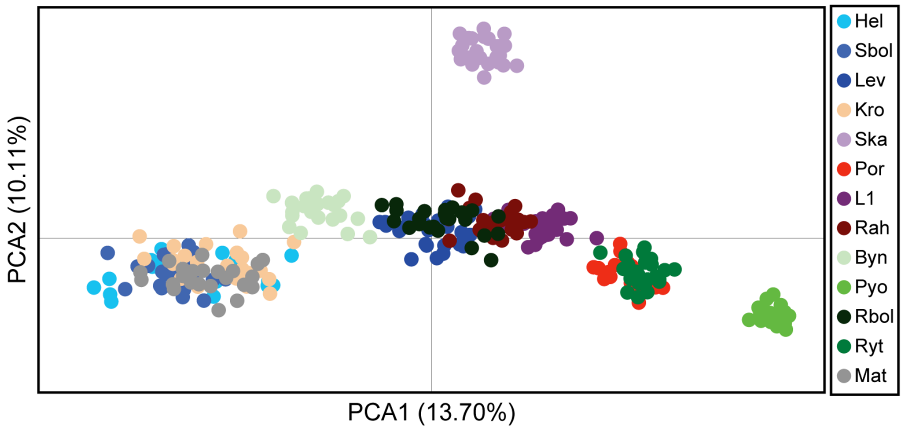

Figure S1 Principal component analysis (PCA) of 312 nine-spined stickleback individuals from 13 different populations based on 109 microsatellite loci. Small colored circles represent individuals and the used colors correspond to those in Fig 1. The population abbreviations are defined in Table 1.
