# Supplementary material for: Genome-Wide Linkage Disequilibrium in Nine-Spined Stickleback Populations
Source: G3 (Bethesda). 2014 Aug 12;4(10):1919–29. doi: 10.1534/g3.114.013334 (PMC4199698; doi:10.1534/g3.114.013334)
Supplement: Supporting Information [file supp_g3.114.013334_FigureS2.pdf]

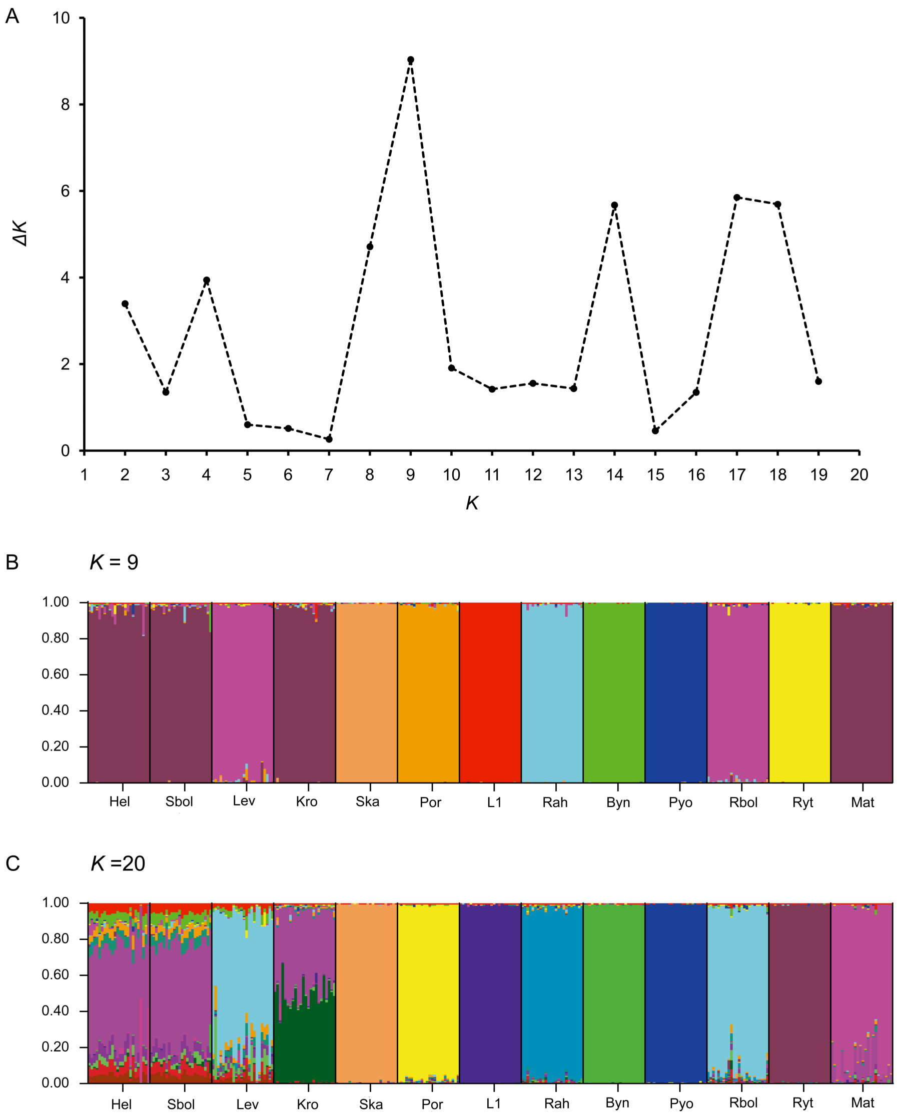

Figure S2 STRUCTURE outputs for joint analysis of the 13 nine-spined stickleback populations. (A) The maximum value of ΔK indicates the most likely number of genetic clusters (K) was nine. (B) Individual membership bar plot of the optimal nine genetic clusters. (C) Individual membership bar plot of the maximum tested 20 genetic clusters. The genotype of each individual is represented by a thin vertical bar representing the membership proportions in each of the genetic clusters. Each colour stands for a genetic cluster. No substructure was found within any of the populations at both K = 9 and K = 20. The population abbreviations are defined in Table 1.
