# Supplementary material for: Genome-Wide Linkage Disequilibrium in Nine-Spined Stickleback Populations
Source: G3 (Bethesda). 2014 Aug 12;4(10):1919–29. doi: 10.1534/g3.114.013334 (PMC4199698; doi:10.1534/g3.114.013334)
Supplement: Supporting Information [file supp_g3.114.013334_FigureS3.pdf]

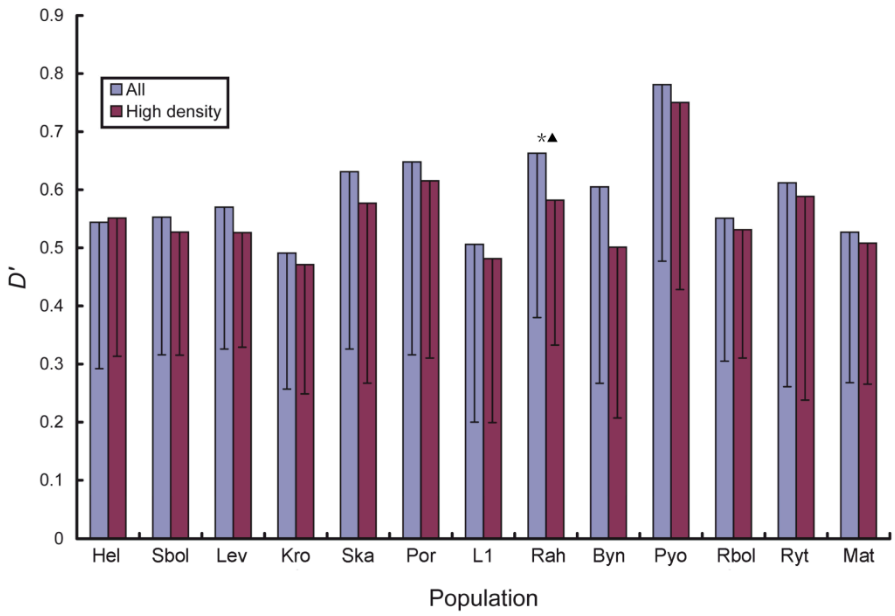

Figure S3 Histograms showing comparisons of mean *D′* values based on all 109 microsatellite markers (genome-wide) and on 38 microsatellite markers in four LGs with highest density of markers in 13 nine-spined stickleback populations. The line within vertical bar represents standard deviation of the mean value. Significant differences in *D′* values are indicated by asterisk (Mann-Whitney U tests, $P < 0.05$) and triangle (ANCOVAs, $P < 0.05$).
