# Supplementary material for: Genome-Wide Linkage Disequilibrium in Nine-Spined Stickleback Populations
Source: G3 (Bethesda). 2014 Aug 12;4(10):1919–29. doi: 10.1534/g3.114.013334 (PMC4199698; doi:10.1534/g3.114.013334)
Supplement: Supporting Information [file supp_g3.114.013334_TableS1.pdf]

Table S1 The number and proportion of rare alleles (allele frequency < 0.05) in 13 nine-spined stickleback populations using 109 microsatellite markers.

Population	Total number of alleles	The number of rare alleles	Proportion of rare alleles
Hel (M)	760	399	0.53
Sbol (M)	757	392	0.52
Lev (M)	765	395	0.52
Kro (L)	647	267	0.41
Ska (L)	266	45	0.17
Por (L)	397	159	0.40
L1 (L)	266	68	0.26
Rah (L)	524	245	0.47
Byn (P)	240	39	0.16
Pyo (P)	164	25	0.15
Rbol (P)	656	299	0.46
Ryt (P)	253	62	0.25
Mat (R)	507	146	0.29

M, marine; L, lake; P, pond; R, river. The population abbreviations are defined in Table 1.
