# Supplementary material for: Genome-Wide Linkage Disequilibrium in Nine-Spined Stickleback Populations
Source: G3 (Bethesda). 2014 Aug 12;4(10):1919–29. doi: 10.1534/g3.114.013334 (PMC4199698; doi:10.1534/g3.114.013334)
Supplement: Supporting Information [file supp_g3.114.013334_TableS2.pdf]

Table S2 Matrix of pairwise F_{ST} estimates (lower diagonal) and their statistical significance (upper diagonal) between 13 nine-spined stickleback populations based on 109 microsatellite loci.

Populations	Hel	Sbol	Lev	Kro	Ska	Por	L1	Rah	Byn	Pyo	Rbol	Ryt	Mat
Hel	—	NS	*	*	NS	NS	*	NS	*	*	*	*	*
Sbol	0.003	—	NS	NS	NS	NS	*	NS	*	*	*	*	*
Lev	0.100	0.103	—	*	NS	*	*	NS	*	*	*	*	*
Kro	0.020	0.017	0.096	—	NS	*	*	NS	*	*	*	*	*
Ska	0.376	0.372	0.316	0.359	—	NS	NS	NS	NS	NS	NS	NS	NS
Por	0.278	0.279	0.227	0.265	0.521	—	*	NS	*	*	*	*	*
L1	0.291	0.296	0.241	0.288	0.527	0.405	—	NS	*	*	*	*	*
Rah	0.243	0.248	0.195	0.239	0.475	0.390	0.388	—	NS	*	NS	*	NS
Byn	0.326	0.324	0.339	0.336	0.589	0.486	0.523	0.477	—	*	*	*	*
Pyo	0.455	0.455	0.410	0.449	0.724	0.548	0.617	0.573	0.703	—	*	*	*
Rbol	0.110	0.113	0.018	0.105	0.312	0.236	0.254	0.190	0.346	0.413	—	*	*
Ryt	0.352	0.362	0.314	0.357	0.604	0.454	0.492	0.428	0.571	0.679	0.314	—	*
Mat	0.044	0.043	0.120	0.057	0.398	0.304	0.311	0.278	0.347	0.472	0.130	0.371	—

NS, not significant; *Statistical significance at Bonferroni adjusted ($P < 0.000641$) alpha level of $P = 0.05$. The population abbreviations are defined in Table 1.
