# Supplementary material for: Genome-Wide Linkage Disequilibrium in Nine-Spined Stickleback Populations
Source: G3 (Bethesda). 2014 Aug 12;4(10):1919–29. doi: 10.1534/g3.114.013334 (PMC4199698; doi:10.1534/g3.114.013334)
Supplement: Supporting Information [file supp_g3.114.013334_TableS3.pdf]

Table S3 Results of bottleneck tests in 13 nine-spined stickleback populations form software Bottleneck under TPM mutation model with a one-tailed Wilcoxon signed-rank tests (*P* values) and from *M*-ratio tests (*M* and M_c values) using 109 microsatellite markers.

Population	TPM	*M*	M_c
Hel	1.00	0.693	0.874*/0.792*/0.755*
Sbol	1.00	0.736	0.874*/0.792*/0.755*
Lev	1.00	0.753	0.874*/0.792*/0.755*
Kro	0.92	0.714	0.874*/0.792*/0.755*
Ska	0.89	0.847	0.906*/0.884*/0.861*
Por	1.00	0.797	0.916*/0.893*/0.871*
L1	0.03*	0.705	0.914*/0.891*/0.869*
Rah	1.00	0.780	0.915*/0.893*/0.872*
Byn	0.42	0.806	0.911*/0.889*/0.866*
Pyo	0.96	0.898	0.896/0.872/0.849
Rbol	1.00	0.735	0.874*/0.791*/0.755*
Ryt	0.69	0.772	0.911*/0.889*/0.867*
Mat	0.47	0.670	0.874*/0.792*/0.755*

*$P < 0.05$. The three M_c values from left to right correspond to the tests of a pre-bottleneck N_e of 1,000, 5,000, 10,000 for the three marine (Hel, Sbol, Lev) and three coastal freshwater (Kro, Rbol, Mat) populations, and 100, 500 and 1000 for the seven inland freshwater populations (Ska, Por, L1, Rah, Byn, Pyo, Rbol, Ryt), respectively.
