# Supplementary material for: Genome-Wide Linkage Disequilibrium in Nine-Spined Stickleback Populations
Source: G3 (Bethesda). 2014 Aug 12;4(10):1919–29. doi: 10.1534/g3.114.013334 (PMC4199698; doi:10.1534/g3.114.013334)
Supplement: Supporting Information [file supp_g3.114.013334_TableS4.pdf]

Table S4 Linkage disequilibrium estimates (r^2 ± S.E.) for syntenic markers in nine-spined stickleback populations and habitat types using 109 microsatellite markers.

Data Set	Physical distance interval (Syntenic)					Overall
	0-5 Mb	5-10 Mb	10-15 Mb	15-20 Mb	>20 Mb	(Syntenic)
Hel (M)	0.031 (0.038)	0.032 (0.034)	0.028 (0.024)	0.024 (0.017)	0.037 (0.013)	0.030 (0.033)
Sbol (M)	0.035 (0.042)	0.033 (0.038)	0.033 (0.033)	0.029 (0.014)	0.020 (0.010)	0.033 (0.037)
Lev (M)	0.026 (0.018)	0.038 (0.066)	0.032 (0.032)	0.041 (0.030)	0.028 (0.018)	0.032 (0.043)
Kro (L)	0.033 (0.028)	0.035 (0.042)	0.026 (0.022)	0.022 (0.013)	0.022 (0.011)	0.032 (0.032)
Ska (L)	0.037 (0.032)	0.048 (0.036)	0.040 (0.041)	0.059 (0.046)	0.022 (0.012)	0.041 (0.035)
Por (L)	0.030 (0.032)	0.030 (0.057)	0.021 (0.027)	0.035 (0.034)	0.034 (0.042)	0.030 (0.042)
L1 (L)	0.052 (0.062)	0.062 (0.158)	0.085 (0.148)	0.034 (0.028)	0.011 (0.011)	0.056 (0.107)
Rah (L)	0.036 (0.057)	0.034 (0.094)	0.037 (0.043)	0.033 (0.038)	0.082 (0.065)	0.036 (0.069)
Byn (P)	0.035 (0.035)	0.066 (0.097)	0.077 (0.121)	0.034 (0.031)	0.015 (0.009)	0.050 (0.076)
Pyo (P)	0.045 (0.044)	0.131 (0.307)	0.008 (0.008)	0.013 (0.004)	—	0.065 (0.167)
Rbol (P)	0.029 (0.026)	0.045 (0.084)	0.034 (0.033)	0.027 (0.014)	0.029 (0.012)	0.035 (0.054)
Ryt (P)	0.046 (0.062)	0.049 (0.144)	0.036 (0.046)	0.047 (0.035)	0.013 (0.010)	0.046 (0.094)
Mat (R)	0.041 (0.049)	0.038 (0.042)	0.046 (0.051)	0.031 (0.022)	0.035 (0.022)	0.040 (0.045)
Marine (average[a])	0.031 (0.005)	0.034 (0.003)	0.031 (0.003)	0.031 (0.009)	0.028 (0.009)	0.032 (0.002)
Lake (average[a])	0.038 (0.009)	0.042 (0.013)	0.042 (0.025)	0.037 (0.014)	0.034 (0.028)	0.039 (0.010)
Pond (average[a])	0.039 (0.008)	0.073 (0.040)	0.039 (0.029)	0.030 (0.014)	0.019 (0.009)	0.049 (0.012)
CF (average[a])	0.034 (0.006)	0.039 (0.005)	0.035 (0.010)	0.027 (0.005)	0.029 (0.007)	0.036 (0.004)
Marine (combined[b])	0.014 (0.017)	0.016 (0.024)	0.015 (0.017)	0.012 (0.013)	0.010 (0.008)	0.014 (0.019)
Lake (combined[b])	0.035 (0.043)	0.035 (0.066)	0.036 (0.044)	0.034 (0.031)	0.021 (0.024)	0.035 (0.051)
Pond (combined[b])	0.057 (0.073)	0.069 (0.087)	0.068 (0.088)	0.085 (0.129)	0.042 (0.058)	0.064 (0.084)
CF (combined[b])	0.024 (0.043)	0.020 (0.022)	0.026 (0.023)	0.018 (0.011)	0.014 (0.010)	0.022 (0.033)
River	0.041 (0.049)	0.038 (0.042)	0.046 (0.051)	0.031 (0.022)	0.035 (0.022)	0.040 (0.045)

M, marine; L, lake; P, pond; R, river; CF, Coastal freshwater, including Kro, Rbol and Mat. The population abbreviations are defined in Table 1.

[a] r^2 value is obtained from the averaged r^2 value of relevant populations.

[b] r^2 value is calculated from the combined original haplotype data of relevant populations.
