# Supplementary material for: Genome-Wide Linkage Disequilibrium in Nine-Spined Stickleback Populations
Source: G3 (Bethesda). 2014 Aug 12;4(10):1919–29. doi: 10.1534/g3.114.013334 (PMC4199698; doi:10.1534/g3.114.013334)
Supplement: Supporting Information [file supp_g3.114.013334_TableS5.pdf]

Table S5 Results of Pearson's and Kendall's correlation tests between *D'* and r^2 values in nine-spined stickleback populations and habitat types.

Population	Pearson's correlation coefficient	Kendall's correlation coefficient
Hel (M)	0.178**	0.277**
Sbol (M)	0.180**	0.175**
Lev (M)	0.265**	0.263**
Kro (L)	0.291**	0.307**
Ska (L)	0.160	0.175*
Por (L)	0.117	0.105*
L1 (L)	0.317**	0.371**
Rah (L)	0.149*	0.081
Byn (P)	0.244**	0.265**
Pyo (P)	0.106	-0.146
Rbol (P)	0.231**	0.279**
Ryt (P)	0.176*	0.218**
Mat (R)	0.263**	0.282**
Marine (combined data)	0.172**	0.195**
Lake (combined data)	0.274**	0.234**
Pond (combined data)	0.449**	0.417**
CF (combined data)	0.286**	0.304**
River	0.263**	0.282**

**P* < 0.05, ***P* < 0.01. M, marine; L, lake; P, pond; R, river; CF, Coastal freshwater, including Kro, Rbol and Mat. The population abbreviations are defined in Table 1.
